# Sooty mangabey genome sequence provides insight into AIDS resistance in a natural SIV host

**DOI:** 10.1038/nature25140

**Published:** 2018-01-03

**Authors:** David Palesch, Steven E. Bosinger, Gregory K. Tharp, Thomas H. Vanderford, Mirko Paiardini, Ann Chahroudi, Zachary P. Johnson, Frank Kirchhoff, Beatrice H. Hahn, Robert B. Norgren, Nirav B. Patel, Donald L. Sodora, Reem A. Dawoud, Caro-Beth Stewart, Sara M. Seepo, R. Alan Harris, Yue Liu, Muthuswamy Raveendran, Yi Han, Adam English, Gregg W. C. Thomas, Matthew W. Hahn, Lenore Pipes, Christopher E. Mason, Donna M. Muzny, Richard A. Gibbs, Daniel Sauter, Kim Worley, Jeffrey Rogers, Guido Silvestri

**Affiliations:** 1 Emory Vaccine Center and Yerkes National Primate Research Center, Emory University, Atlanta, Georgia 30329, USA; 2 Department of Pathology and Laboratory Medicine, Emory University School of Medicine, Atlanta, Georgia 30329, USA; 3 Department of Pediatrics, Emory University School of Medicine, Atlanta, Georgia 30329, USA; 4 Institute of Molecular Virology, Ulm University Medical Center, 89081 Ulm, Germany; 5 Departments of Medicine and Microbiology, Perelman School of Medicine, University of Pennsylvania, Philadelphia, Pennsylvania 19104, USA; 6 Department of Genetics, Cell Biology and Anatomy, University of Nebraska, Medical Center, Omaha, Nebraska 68198, USA; 7 Center for Infectious Disease Research, formerly Seattle Biomedical Research Institute, Seattle, Washington 98109, USA; 8 Department of Biological Sciences, University at Albany-State University of New York, Albany, New York 12222, USA; 9 Human Genome Sequencing Center, Baylor College of Medicine, Houston, Texas 77030, USA; 10 Department of Molecular and Human Genetics, Baylor College of Medicine, Houston, Texas 77030, USA; 11 Department of Biology and School of Informatics and Computing, Indiana University, Bloomington, Indiana 47405, USA; 12 Department of Physiology and Biophysics, Weill Cornell Medical College, New York, New York 10065, USA

## Abstract

In contrast to infections with human immunodeficiency virus (HIV) in humans and simian immunodeficiency virus (SIV) in macaques, SIV infection of a natural host, sooty mangabeys (*Cercocebus atys*), is non-pathogenic despite high viraemia^1^. Here we sequenced and assembled the genome of a captive sooty mangabey. We conducted genome-wide comparative analyses of transcript assemblies from *C. atys* and AIDS-susceptible species, such as humans and macaques, to identify candidates for host genetic factors that influence susceptibility. We identified several immune-related genes in the genome of *C. atys* that show substantial sequence divergence from macaques or humans. One of these sequence divergences, a C-terminal frameshift in the toll-like receptor-4 (*TLR4*) gene of *C. atys*, is associated with a blunted *in vitro* response to TLR-4 ligands. In addition, we found a major structural change in exons 3–4 of the immune-regulatory protein intercellular adhesion molecule 2 (ICAM-2); expression of this variant leads to reduced cell surface expression of ICAM-2. These data provide a resource for comparative genomic studies of HIV and/or SIV pathogenesis and may help to elucidate the mechanisms by which SIV-infected sooty mangabeys avoid AIDS.

SIV infection of natural hosts, such as sooty mangabeys, is typically non-pathogenic despite high viraemia. This is in stark contrast to HIV infection in humans and experimental SIV infection in rhesus macaques (*Macaca mulatta*) that progress to AIDS unless treated with antiretroviral therapy. The main virological and immunological features of natural SIV infection in sooty mangabeys have been described over the past 15 years in studies that compared and contrasted this infection with the pathogenic infections of HIV and SIV in humans and rhesus macaques^1^. SIV-infected sooty mangabeys show several features that have been observed in pathogenic infections, including high viraemia, short *in vivo* lifespan of productively infected cells, depletion of mucosal CD4^+^ T cells, strong type-I interferon response in the acute infection, and cellular immune responses that fail to control virus replication. However, in contrast to pathogenic infections, SIV-infected sooty mangabeys (i) have healthy CD4^+^ T cell levels; (ii) do not experience mucosal immune dysfunction, avoiding depletion of T helper 17 (T_H_17) cells, intestinal epithelial damage and microbial translocation; (iii) maintain low levels of immune activation during the chronic infection; and (iv) achieve compartmentalization of virus replication that preserves central-memory and stem-cell memory CD4^+^ T cells as well as follicular T_H_ cells^1,2^. An additional notable feature of SIV infection in natural hosts is the low rate of mother-to-infant transmission that is related to low expression of CCR5 on circulating and mucosal CD4^+^ T cells^3^. Although many aspects of the natural course of SIV infection in sooty mangabeys have now been described, the key molecular mechanisms by which these animals avoid AIDS remain poorly understood.

In this study, we sequenced the genome of a captive sooty mangabey and compared this genome to the genomes of AIDS-susceptible primates to look for candidate genes that may influence susceptibility to AIDS in SIV-infected hosts. We sequenced genomic DNA to a whole-genome coverage of about 180× using the Illumina HiSeq 2000 platform, and produced an initial assembly using ALLPATHS-LG, Atlas-Link and Atlas-GapFill (see Methods for details). The total size of the assembled *C. atys* genome (Caty_1.0; NCBI accession number GCA_000955945.1) is around 2.85 Gb, with a contig N50 size of 112.9 kb and scaffold N50 size of 12.85 Mb (Table 1). Genome annotation identified 20,829 protein-coding genes and 4,464 non-coding genes in the *C. atys* assembly, which is comparable to other available draft quality genomes of nonhuman primates (Table 1). These analyses demonstrate that the Caty_1.0 reference genome is of sufficient quality to facilitate population-scale whole-genome and transcriptome sequencing studies.

To identify novel immunogenetic factors specific to *C. atys* that may be involved in the ability of this species to avoid progression to AIDS, we established a bioinformatic pipeline for a comparative protein analysis (Fig. 1 and Extended Data Fig. 1, see Methods for details). Using this approach, we found 34 candidate immune-related genes with sequences that diverged between *C. atys* and *M. mulatta* (Table 1 and Extended Data Table 1). Although we cannot exclude a role of immune genes with minor differences in *C. atys* and *M. mulatta*, the highly divergent genes listed in Table 1 and Extended Data Table 1 constitute candidate genes involved in the outcomes of SIV infection in these two species.

Our screen identified sequence divergence in a number of proteins that are important during HIV infection, such as APOBEC3C (91.6%) and BST2 (also known as tetherin, 95.1%), as well as pattern-recognition receptors (MBL2, CLEC4A, CLEC4D and CLEC6A), the antiviral sensor cyclic GMP–AMP synthase (cGAS (also known as MB21D1)) and other immune mediators (Extended Data Table 1). Because CD4 and CCR5 are important for AIDS pathogenesis, we aligned the sequences of *Ca*CD4 and *Ca*CCR5 to *Mm*CD4 and *Mm*CCR5, respectively^4,5^. Neither gene showed any major structural changes in the wild-type variants, although CD4 was slightly below the 97% threshold of identity (Extended Data Fig. 1b, c). In addition, we found specific gene families in *C. atys* that are expanded relative to *M. mulatta*, humans and other primates (Extended Data Table 2a). Notably, we detected localized regions of increased substitution, defined by a clustered difference of three or more amino acids, in 10 genes. The most marked variations in the amino acid sequence of *C. atys* compared to *M. mulatta* were observed in ICAM-2 and TLR-4 (Table 1).

ICAM-2 is an approximately 60-kDa transmembrane glycoprotein of the immunoglobulin superfamily, which is expressed on various immune cells and implicated in lymphocyte homing and recirculation^6^. ICAM-2 ligands are lymphocyte function-associated antigen-1 and the C-type lectin DC-SIGN^7^. We discovered a misalignment of the ICAM-2 proteins between *C. atys* and *M. mulatta* that starts in exon 3 (Extended Data Fig. 2a). This difference is explained by a 499-bp deletion starting from exon 3 of Ca*ICAM2*, as detected by PCR and Sanger sequencing (Fig. 2a and Extended Data Fig. 3). We subsequently confirmed the expression of this truncated form of ICAM-2 in ten out of ten additional *C. atys* genome sequences (Extended Data Fig. 2b). By contrast, analysis of the whole-genome sequences of 15 baboons and more than 130 rhesus macaques demonstrated that only the full-length ICAM-2 protein was found in all individuals (data not shown)^8^. The ICAM-2 deletion may be specific to *C. atys*, as it is not present in any other known primate sequences, including other natural SIV hosts, such as the African green monkey, drill and colobus monkey. Transcript models generated from *de novo* assembled *C. atys* RNA-sequencing (RNA-seq) data from 14 different tissues showed that the mature mRNA sequence of Ca*ICAM2* retains substantial portions of what is part of the intronic sequence in other nonhuman primates, and thus codes for a markedly different final gene product (Extended Data Figs 2, 3). Splice-junction sequence analysis showed intact splicing for all four exons in *M. mulatta*, but no splice junctions were found between exons 3 and 4 in *C. atys*, indicating severe splicing defects due to the deletion (Extended Data Fig. 4).

To test whether the observed genetic difference in *ICAM2* has functional consequences, we measured ICAM-2 surface expression on immune cells from humans, *M. mulatta* and *C. atys* with an antibody that recognizes a conserved epitope between these species^9^. ICAM-2 was readily detected on T cells and B cells from humans and *M. mulatta*, but not from *C. atys* (Fig. 2b, c), suggesting that ICAM-2 is not functional in lymphocytes of *C. atys*. However, a truncated, lower molecular weight form of ICAM-2 could be detected intracellularly by western blot in *C. atys* cells (Fig. 2d), thus demonstrating the presence of the predicted truncated ICAM-2 protein. Overall, these data indicate that the presence of a species-specific gene sequence difference in Ca*ICAM2* results in the abrogation of surface expression of this protein in *C. atys*. Further studies are needed to elucidate potential links between this truncated form of ICAM-2 and the remarkable immunological features of SIV infection in this species.

TLR-4 is a pattern recognition receptor that senses lipopolysaccharides (LPS) on gram-negative bacteria and initiates pro-inflammatory cytokine induction, maturation and activation in macrophages, dendritic cells and other immune cells. During pathogenic HIV or SIV infections, exacerbated TLR-4 stimulation and concomitant proinflammatory signalling elicited by microbial translocation is considered a primary mechanism that underlies HIV-induced chronic immune activation^10,11^. Here, we found that the TLR-4 protein sequences of *M. mulatta* and *C. atys* are markedly different at the C terminus (Extended Data Fig. 5a). We confirmed the underlying difference in the *TLR4* nucleotide sequence by Sanger sequencing (Extended Data Fig. 5b, c). We next analysed the genomic DNA sequence of *TLR4* in 10 additional sooty mangabeys and found that the observed DNA sequence difference was present in all individuals (Extended Data Fig. 6a). Alignment of TLR-4 protein sequences from different primate species revealed that the 17-amino-acid longer C-terminal sequence is only found in natural SIV hosts, such as African green monkey, drill and colobus monkey (Fig. 3a), whereas non-natural hosts, including *M. mulatta* and baboons show expression of the short TLR-4 C-terminal sequence.

The divergence of TLR-4 amino acid sequences amongst Old World primates shows an interesting pattern of molecular evolution. First, the genomic sequence encoding the *TLR4* C terminus is defined by a 1-bp deletion causing a frame shift in all Old World monkeys, both natural and non-natural hosts, including colobine and cercopithecine lineages, but it is not found in either hominoids (apes and humans) or platyrrhines (New World monkeys) (Extended Data Fig. 6b). This suggests that this mutation occurred after the hominoid–Old World monkey divergence approximately 25 million years ago^12^. Second, there is a G-to-A nucleotide substitution in the non-natural host Old World monkeys (baboons and macaques) that creates a truncated protein in these species^8^ (Extended Data Fig. 6b). Although a naive analysis of this pattern would suggest two independent mutational changes in *TLR4*, the short internal branch of the species tree implies that incomplete lineage sorting of an ancestral polymorphism could also generate this pattern^13^ (Fig. 3b). To test this hypothesis, we examined the *TLR4* gene tree among 17 primate species. While generally supporting the relationships among these species (Fig. 3b), the analysis also found a number of nucleotide positions—spaced throughout the gene—consistent with incomplete lineage sorting between *C. atys*, baboon and *M. mulatta* (Extended Data Fig. 7). The incomplete lineage sorting hypothesis is also more likely, given that balancing selection is often found to be acting on immune-related genes. Therefore, even though baboons are believed to be more closely related to sooty mangabeys and drills than to rhesus macaques, the phylogeny of Old World monkeys is compatible with the possibility of a single G-to-A mutation creating the truncated form of the protein in the common ancestor of baboons, rhesus macaques and sooty mangabeys^12,14^ (Fig. 3b).

We next investigated potential differences in TLR-4 function between *M. mulatta* and *C. atys*. Our previous work has shown that macrophages from *C. atys* exhibit higher expression of tetherin, APOBEC and TRIM5α in response to LPS compared to *M. mulatta*^15^. This is consistent with the relative resistance of *C. atys* macrophages to *in vivo* SIV infection after experimental CD4^+^ T cell depletion compared to SIV-infected *M. mulatta* macrophages^16^. Here we analysed cytokine gene expression and protein production after LPS stimulation, and found reduced mRNA expression and secretion of TNF (also known as TNF-α) and IL-6 in cells from *C. atys* compared to *M. mulatta* (Fig. 3c, d). Because some commercial LPS preparations contain lipoprotein contaminants that can induce TLR-2 signalling, we confirmed the TLR-4 specificity of the reduced LPS response using the selective TLR-4 agonist^17^ lipid-A (Extended Data Fig. 8a, b). Next, we found that the species-specific differences between *C. atys* and *M. mulatta* in LPS-induced TNF and IL-6 production were maintained in acute and chronic infection (Fig. 3e and Extended Data Fig. 8c). Additionally, we did not observe any difference in the mRNA levels of *TLR4* in cells from *C. atys* and *M. mulatta*, nor did the expression of any factors in the TLR-4–MyD88–TRIF signalling axis correlate with TNF and IL-6 production (Extended Data Fig. 8d and Extended Data Table 3). To more broadly characterize the effect of attenuated TLR-4 signalling in *C. atys*, we performed comparative RNA-seq profiling of LPS-treated monocytes, and found lower production of Ca*TNF* and Ca*IL6* mRNA (Extended Data Fig. 8e). Moreover, using gene set enrichment analysis (GSEA), we observed that induction of pro-inflammatory genes was broadly and significantly reduced in cells from *C. atys* (Fig. 3f, g and Extended Data Fig. 9). Overall, these results indicate that LPS stimulation of blood cells from *C. atys* results in a blunted production of pro-inflammatory cytokines. To establish a link between the C-terminal *TLR4* sequence difference and the responsiveness to LPS, we analysed the TLR-4 orthologues of humans, *C. atys* and *M. mulatta* in an NF-κB reporter assay. We observed a significantly attenuated NF-κB response to LPS of *C. atys* TLR-4 (*Ca*TLR-4) compared to *M. mulatta* TLR-4 (*Mm*TLR-4). Using chimaeric constructs encoding Mm*TLR4* with the C terminus of Ca*TLR4* or Ca*TLR4* with the C terminus of *Mm*TLR4, we confirmed that the TLR4 C terminus is responsible for this phenotypic difference (Fig. 3h). This demonstrates a sequence–function relationship of the TLR4 C terminus and suggests a novel mechanism contributing to the lower immune activation of SIV-infected sooty mangabeys.

Over the past decade the genomes of more than 25 nonhuman primate species have been sequenced, assembled and annotated^18^. This knowledge has improved our understanding of primate evolution, biology and general physiology, which has informed human biology and medicine. Here, we report a high-coverage, high-contiguity whole-genome sequence for *C. atys*, a natural SIV host. Comparative genomic analyses of natural and non-natural SIV hosts provide candidate genes that potentially influence susceptibility to AIDS in SIV-infected hosts. We have previously used trancriptomics to characterize the host response to SIV infection of *C. atys* and African green monkeys^19,20^. Here, we examined the mechanisms of AIDS resistance of a natural SIV host genome-wide using genome sequencing. We identified candidate genes that show sequence changes that are specific to *C. atys* and two gene products (ICAM-2 and TLR-4), which show structural differences between *C. atys* and *M. mulatta* that may influence cell-surface expression (ICAM-2) and downstream signalling (TLR-4) of these proteins. Our findings may also explain prior results showing that not all natural SIV hosts respond to infection in the same way, suggesting that in each primate species, multiple distinct mechanisms may contribute to the phenotype, rather than mutations in single genes, as has been purported, and eventually refuted, in other studies^21^. Further comparative studies with additional natural SIV host species may identify additional similarities (or differences) in the genes involved in the evolutionary pathways that led to AIDS resistance in different species of African nonhuman primates.

In this study, we used whole-genome sequencing and comparative genomic analysis to identify candidate genes regulating host resistance to AIDS. Future studies in which these candidate genes are manipulated *in vivo* during SIV infection are needed to characterize to what extent these genes may influence the non-pathogenic nature of SIV infection in sooty mangabeys.

## Online Content

Methods, along with any additional Extended Data display items and Source Data, are available in the online version of the paper; references unique to these sections appear only in the online paper.

## Methods

### Sequencing and assembly of the sooty mangabey genome

DNA from a female sooty mangabey (*C. atys*) born and maintained at the Yerkes National Primate Research Center was extracted from whole blood. The animal selected for sequencing was one of the original dams of a large matrilineal line of the colony. In addition, she possessed the most common MHC haplotype observed within the group. As such, her genetic constitution within the closed population was thought to be the most representative of any single animal. All animals were housed at the Yerkes National Primate Research Center of Emory University and maintained in accordance with US NIH guidelines. All studies were approved by the Emory University Institutional Animal Care and Usage Committee. Following quality control to ensure purity and molecular weight, a series of Illumina sequencing libraries were prepared using standard procedures. Paired-end libraries with nominal insert sizes 180 bp and 500 bp were produced. In brief, 1 µg of DNA was sheared to the desired size using a Covaris S-2 system. Sheared fragments were purified with Agencourt AMPure XP beads, end-repaired, dA-tailed and ligated to Illumina universal adaptors. After adaptor ligation, DNA fragments were further size selected by agarose gel and PCR amplified for six to eight cycles using Illumina P1 and Index primer pair and Phusion High-Fidelity PCR Master Mix (New England Biolabs). The final library was purified using Agencourt AMPure XP beads and quality assessed by Agilent Bioanalyzer 2100 (DNA 7500 kit) to determine library quantity and fragment size distribution before sequencing.

Long mate-pair libraries with 2-kb, 3-kb, 5-kb and 8-kb insert sizes were constructed according to the manufacturer’s protocol (Mate Pair Library v.2 Sample Preparation Guide 15001464 Rev. A Pilot Release). In brief, 5 µg (for 2- and 3-kb size libraries) or 10 µg (5- and 8-kb libraries) of genomic DNA was sheared to the desired size by Hydroshear (Digilab), then end-repaired and biotinylated. Fragment sizes between 1.8–2.5 kb (2 kb), 3.0–3.7 kb (3 kb), 4.5–6.0 kb (5 kb) or 8–10 kb (8 kb) were purified from a 1% low-melting agarose gel and circularized by blunt-end ligation. These size-selected circular DNA fragments were then sheared to 400 bp (Covaris S-2), purified using Dynabeads M-280 Streptavidin Magnetic Beads, end-repaired, dA-tailed and ligated to Illumina PE sequencing adapters. DNA fragments with adaptor molecules on both ends were amplified for 12 to 15 cycles with Illumina P1 and Index primers. Amplified DNA fragments were purified with Agencourt AMPure XP beads. Quantification and size distribution of the final library was determined as described above before sequencing.

Sequencing was performed on Illumina HiSeq 2000 instruments, generating 100-bp paired-end reads. Raw sequences have been deposited in NCBI under Bioproject PRJNA157077. Reads were assembled using ALLPATHS-LG and further scaffolded and gap-filled using in-house tools Atlas-Link (v.1.0) and Atlas GapFill (v.2.2) (https://www.hgsc.bcm.edu/software/)^23^. Atlas-link is a scaffolding or super-scaffolding method that uses all unused mate pairs to increase scaffold sizes and create new scaffolds in draft-quality assemblies. Those modified scaffolds are then ordered and oriented. Atlas GapFill is run on a super-scaffolded assembly. Regions with gaps are identified and reads mapping within or across those gaps are locally assembled using different assemblers (Phrap, Newbler and Velvet) in order to bridge the gaps with the most conservative assembly of previously unincorporated reads.

PBJelly (v.14.9.9) is a pipeline that improves the contiguity of draft assemblies by filling gaps, increasing contig sizes and super scaffolding by making use of long reads^24^. We used 12.3× coverage of long Pacific Biosciences RSI and RS II sequences, along with the gap-filled Illumina read assembly, as input into PBJelly to produce the final *C. atys* hybrid Illumina–PacBio assembly. This assembly is available at NCBI as Caty1.0 (RefSeq accession GCF_000955945.1).

The total size of the assembled *C. atys* genome is around 2.85 Gb, with a contig N50 size of 112.9 kb and scaffold N50 size of 12.85 Mb (Table 1). By comparison, this contig N50 size is greater than equivalent values for 22 of the 26 other nonhuman primate genome assemblies currently available. To assess completeness, we mapped 21,772 human protein-coding canonical transcripts to Caty_1.0 and found that 94.9% map to this *C. atys* genome with lengths of 95–100% (97.3% of transcripts map at length 70% or greater). As a more stringent test, we mapped 3023 Benchmarking Universal Single-Copy Orthologues (BUSCO) genes and found that over 95% are present in Caty_1.0 (88.8% complete single copy and the others present but duplicated or fragmented)^25^.

Genome annotation was performed through the NCBI Genome Annotation Pipeline, which generated models for genes, transcripts and proteins^26^. To aid accurate transcript annotation, the NCBI pipeline incorporated RNA-seq data from a sooty mangabey pooled tissue reference sample, and data from 14 separate tissues produced through a joint effort by the Nonhuman Primate Reference Transcriptome Resource (NHPRTR; http://www.nhprtr.org/)^27^ and the Human Genome Sequencing Center (HGSC) of Baylor College of Medicine. The NCBI process also used human RefSeq and GenBank transcripts along with other primate protein data.

### Sequencing and polymorphism screen of 10 sooty mangabeys

DNA was prepared from blood or liver samples from 10 sooty mangabeys from the YNPRC colony. Ten sooty mangabey breeder animals were selected in consultation with the YNPRC Breeding Manager representing at least 90% of colony diversity based on the pedigree of the colony. Illumina paired-end libraries (300-bp insert size) were prepared as described above for 500-bp paired-end libraries. These libraries were sequenced (100 bp reads) on a HiSeq2000 instrument, producing an average of 30× whole-genome coverage across individuals. These reads were mapped to the *C. atys* assembly using BWA-mem and single-nucleotide variants were called using GATK (https://software.broadinstitute.org/gatk/). A gVCF file was created for each animal, and variation in the regions of interest for *TLR4* and *ICAM2* were identified in those files.

### Polymorphism screen among rhesus macaques

To assess variation in *TRL4* and *ICAM2* among rhesus macaques, we used our database of whole-genome sequence data from 133 individuals of this species. The details of sequencing and single-nucleotide variants discovery for this population have previously been described^8^. The population-level VCF file for this study was examined for relevant variation in these two genes.

### Targeted re-sequencing of *ICAM2* and *TLR4* in rhesus macaques and sooty mangabeys

To test the validity of the apparent species differences in *ICAM2* and *TLR4* between rhesus macaques and sooty mangabeys, primers were designed to flank three areas of interest (see Extended Data Figs 3a, 5b), PCR was performed using genomic DNA from two rhesus macaques and two sooty mangabeys (including FAK, the animal used for the Caty_1.0 reference genome) and the PCR product was subjected to Sanger sequencing. PCR primers were designed using Primer3 with default settings with the exception that the human mis-priming library was selected (http://bioinfo.ut.ee/primer3/)^28,29^. Primers were tailed with M13 sequences to facilitate Sanger sequencing.

PCR primer pairs (gene specific sequences are underlined): *ICAM2*_Ex2_F GTAAAACGACGGCCAGTATGTGCAGGTGGAGTGTGAT; *ICAM2*_Ex2_R GGAAACAGCTATGACCATGGCTCGAACAGACTCAGTGGA; *ICAM2*_Ex3_F GTAAAACGACGGCCAGTAAGCAGAGCAGGACAGATGT; *ICAM2*_Ex3_R GGAAACAGCTATGACCATGACTCTGCACAGTCAGACCTT; *TLR4*_SL_F GTAAAACGACGGCCAGTACCATGGAATGACTTGCCCT; *TLR4*_SL_R GGAAACAGCTATGACCATGCCTTTCAGCTCTGCCTTCAC.

AmpliTaq Gold 360 DNA Polymerase (Applied Biosystems) was used to amplify PCR products using the following protocol: 95 °C for 10 min; 95 °C for 30 s, 65 °C for 30 s, 72 °C for 30 s, 10 cycles (annealing temperature is decreased by 1 °C per cycle); 94 °C for 30 s, 55 °C for 30 s, 72 °C for 30 s, 30 cycles; 72 °C for 10 min. PCR products were subjected to Sanger sequencing (in both directions) using M13 primers. PCR and Sanger sequencing was performed at ACGT. Traces (see Fig. 2a for examples) were inspected and consensus sequences obtained for each PCR product. Primer sequences were trimmed and consensus sequences were deposited in GenBank (accession numbers: MF468275–MF468286).

### Sequencing and *de novo* assembly of RNA-seq transcripts

Transcripts for sooty mangabey were assembled *de novo* from RNA-seq reads using Trinity on XSEDE’s Blacklight supercomputer^30^. The RNA-seq reads were pooled from 12 different tissues and were prepared by the standard mRNA-seq with the uracil DNA glycosylase protocol (Illumina kit Part RS-122-2303) and are publicly available from the Nonhuman Primate Reference Transcriptome Resource (NCBI SRA accession numbers SRX270666 and SRX270667)^27^. We performed a number of filtering steps to prepare threads for *de novo* assembly, which included removing adapters, filtering for quality, removing poly A/T tails and removing mtDNA and common mammalian rRNA^27,31^. After filtering, we used an input of 1,635,074,685 RNA-seq reads as the basis for the transcriptome assembly. Using around 550 mostly continuous compute hours on Blacklight, we partitioned the computational job into three phases described by the Trinity algorithm: Inchworm (around 100 h × 64 cores), Chrysalis (around 400 h × 128 cores), and Quantify Graph and Butterfly (around 50 h × 64 cores). To circumvent the large amount of I/O generated in the Quantify Graph phase, we ran Trinity directly from the RAM disk for this phase. Using Trinity (version r2012-10-05), the following options were selected: Trinity.pl–JM 512G–no_run_chrysalis–seqType fa–single, reads.fasta–run_as_paired–CPU 16, Trinity.pl–JM 512G–no_run_quantifygraph–seqType fa–single, reads.fasta–run_as_paired–CPU 16–bflyGCThreads 4, Trinity.pl–JM 512G–no_run_butterfly–seqType fa–single reads.fasta–run_as_paired–CPU 16., Trinity.pl–JM 512G–bflyGCThreads 16–bfly-CPU 32–seqType fa, –single reads.fasta–run_as_paired–CPU 16.

The large N25 (6,431 bp), N50 (3,483 bp) and N75 (1,116bp) values of the resulting assembly were indicative of its success.

### Pipeline for finding divergent sooty mangabey proteins

*C. atys* assembly Caty_1.0 protein model predictions were screened against the curated *M. mulatta* MacaM protein models by alignment with BLASTp (v.2.2.28+)^22^. The *C. atys* protein model alignment with the lowest *e* value or highest bitscore (for equal *e* values) was selected for each MacaM protein model, yielding the set of orthologous *C. atys* protein predictions most similar to the *M. mulatta* protein models. The spliced CDS sequence for each Caty_1.0 transcript prediction was extracted with gffread (utility from cufflinks v.2.1.1). Caty_1.0 transcript prediction CDS sequences were screened against the *de novo* RNA-seq assembly transcript models by alignment with BLAT (v.34) and an aligment score was calculated as the number of matching bases minus the number of CDS sequence bases missing in alignment gaps normalized by the CDS sequence length.

**Extended Data Figure 1:**
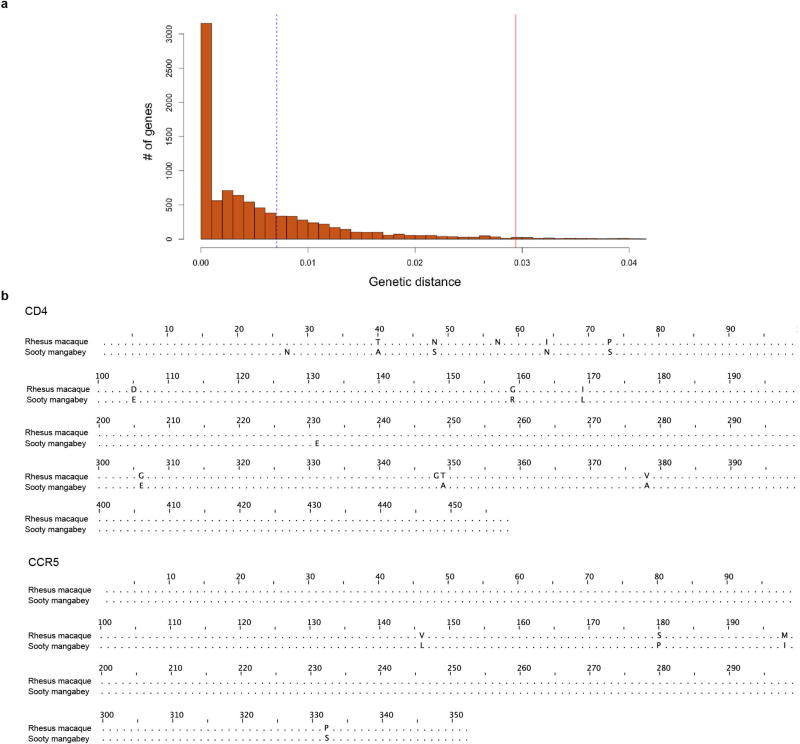
Genetic distances of *C. atys* and *M. mulatta* orthologues and protein sequence alignments of CD4 and CCR5 **a**, Genetic distances of *C. atys* and *M. mulatta* orthologues. The dotted blue line represents a mean distance of 0.00755 expected substitutions, and the solid red line represents the 97th percentile. This percentile indicates that 8,979 out of 9,257 genes have a distance less than 0.0294. **b**, Pairwise alignment of CD4 and CCR5 protein sequences for *C. atys* and *M. mulatta*. Sequences were aligned using Jalview v.2.9.0.

This score penalizes bases missing from the CDS sequence without penalizing extra sequence that may have been added to the RNA-seq transcript model during the assembly process. Only predicted CDS sequences that had a score > 0.99 were retained as supported by RNA-seq data. The MacaM best match selected Caty1.0 protein models were then cross-referenced with the RNA-seq supported Caty_1.0 transcript models to eliminate protein models without RNA-seq evidence. The protein alignments to MacaM for these models were then re-examined to find genes for which the alignment identity was less than 97%, where there were gaps in the alignments or the alignment was not the full length of the protein model. These two species share a common ancestor about 10–11 million years ago, and therefore the expectation is that most proteins will be > 97% identical. This was confirmed by using a maximum likelihood amino acid model (WAG amino acid matrix) to estimate sequence distances between the *C. atys* and *M. mulatta* orthologues (Extended Data Fig. 1). Proteins of interest for differential response to lentivirus infection may be more divergent than expected on average. These represent potentially divergent genes and were further screened against the Gene Ontology (GO) term ‘immune response’. This list of divergent immune genes was then further curated by manual inspection of multiple alignments of cDNA transcript and genomic sequences of *C. atys* (Caty_1.0), *M. mulatta* (MacaM) and human (GRCh38.p7). Multiple alignment analysis was performed using Multalin (http://multalin.toulouse.inra.fr/). *TLR4* and *ICAM2* sequence alignments were generated using Jalview.

### Gene family evolution methods

In order to identify rapidly evolving gene families along the *C. atys* lineage, we obtained peptides from human, chimpanzee, orangutan, gibbon, macaque, baboon, vervet, marmoset and mouse from ENSEMBL 83^32^. The *C. atys* peptides were obtained from NCBI^33^. To ensure that each gene was counted only once, we used only the longest isoform of each protein in each species. We then performed an all-versus-all BLAST search on these filtered sequences^34^. The resulting *e* values from the search were used as the main clustering criterion for the MCL program to group peptides into gene families^35^. This resulted in 14,889 clusters. We then removed all clusters only present in a single species, resulting in 10,967 gene families. We also obtained an ultrametric tree from a previous study and added sooty mangabey based on its divergence time from baboon (TimeTree)^36,37^.

With the gene family data and ultrametric phylogeny as input, we estimated gene gain and loss rates (*λ*) with CAFE v.3.0^38^. This version of CAFE is able to estimate the amount of assembly and annotation error (*ε*) present in the input data using a distribution across the observed gene family counts and a pseudo-likelihood search. CAFE is then able to correct for this error and obtain a more accurate estimate of *λ*. We find an *ε* of about 0.04, which implies that 4% of gene families have observed counts that are not equal to their true counts. After correcting for this error rate, we find *λ* = 0.0020. These values for *ε* and *λ* are on par with those previously reported for mammalian datasets^38,39^ (Extended Data Table 3b). Using the estimated *λ* value, CAFE infers ancestral gene counts and calculates *P* values across the tree for each family and lineage to assess the significance of any gene family changes along a given branch. CAFE uses Monte Carlo re-sampling to assess if a given family is rapidly evolving. For those families found to be rapidly evolving (*P* < 0.01), it then calculates *P* values for each lineage within the family using the Viterbi method. Those lineages with low *P* values (*P* < 0.01) are said to be rapidly evolving.

We observed 1,561 rapidly evolving families across the 10 species of mammals sampled here. Extended Data Table 3c summarizes the gene family changes for all 10 species. Humans have the highest average expansion rate across all families at 0.20 whereas gibbons have the lowest at −0.09, meaning that they have the most gene family contractions. *C. atys* has undergone 535 gene family expansions of which 96 are rapid expansions and 340 gene family contractions of which 48 are rapid contractions.

### Genetic distance between *C. atys* and *M. mulatta* orthologues

**Figure 1:**
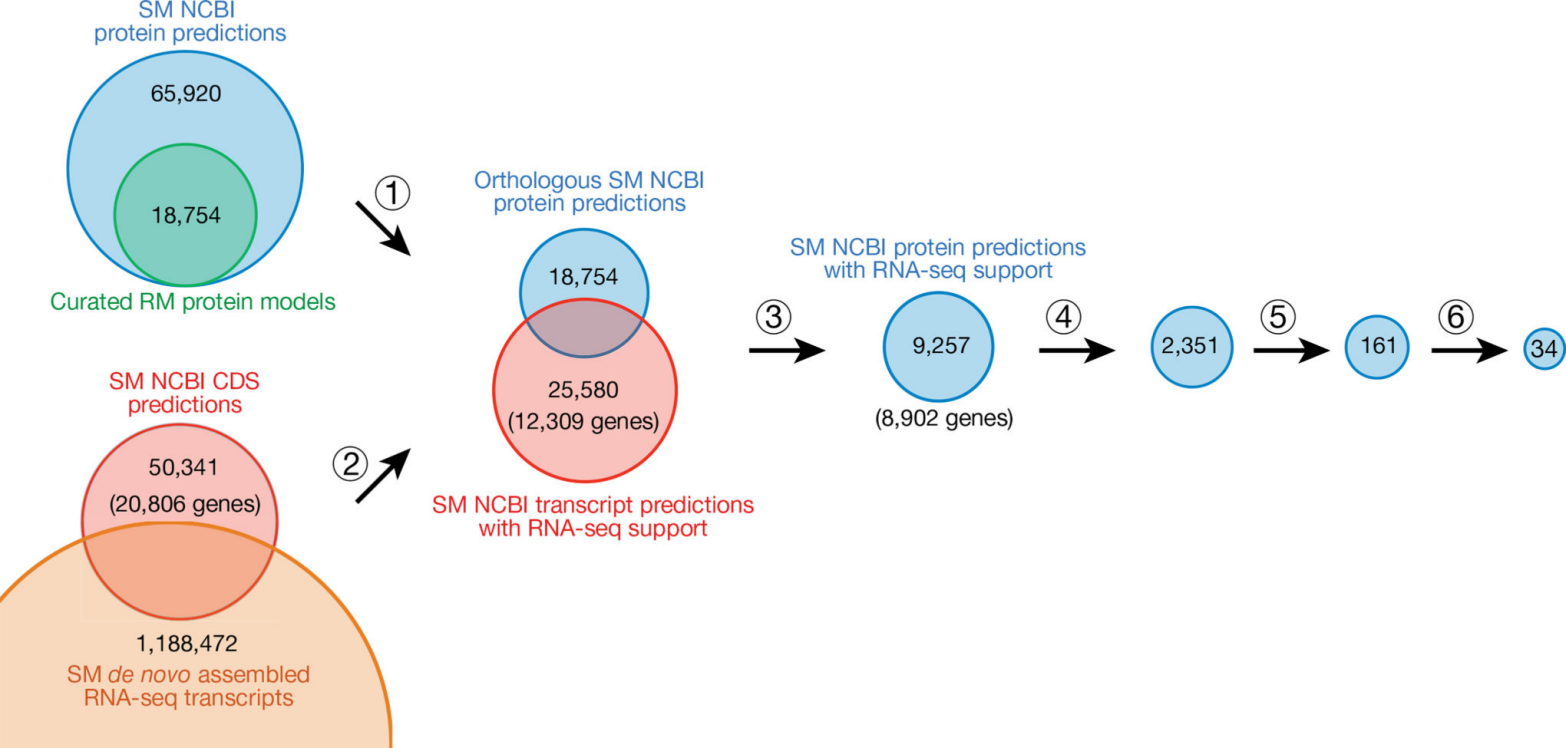
Bioinformatic pipeline for the identification of divergent *C. atys* proteins (1) Sooty mangabey (SM) orthologues were selected by BLAST alignment of *C. atys* NCBI protein predictions (blue) to curated rhesus macaque (RM) protein models (green^22^) and alignment scores were calculated. (2) NCBI transcript predictions with RNA-seq support were identified by BLAT alignment of *de novo* assembled *C. atys* RNA-seq transcripts (orange) to *C. atys* NCBI coding sequence (CDS) predictions (red). (3) Subsquently, corresponding RNA-seq-supported *C. atys* NCBI protein predictions were selected. (4) *C. atys* proteins with high similarity (>97% identity) to *M. mulatta* proteins were filtered out. (5) Immune genes according to Gene Ontology (GO) term classification (immune response) were chosen for further analysis and (6) confirmed by manual inspection.

**Figure 2:**
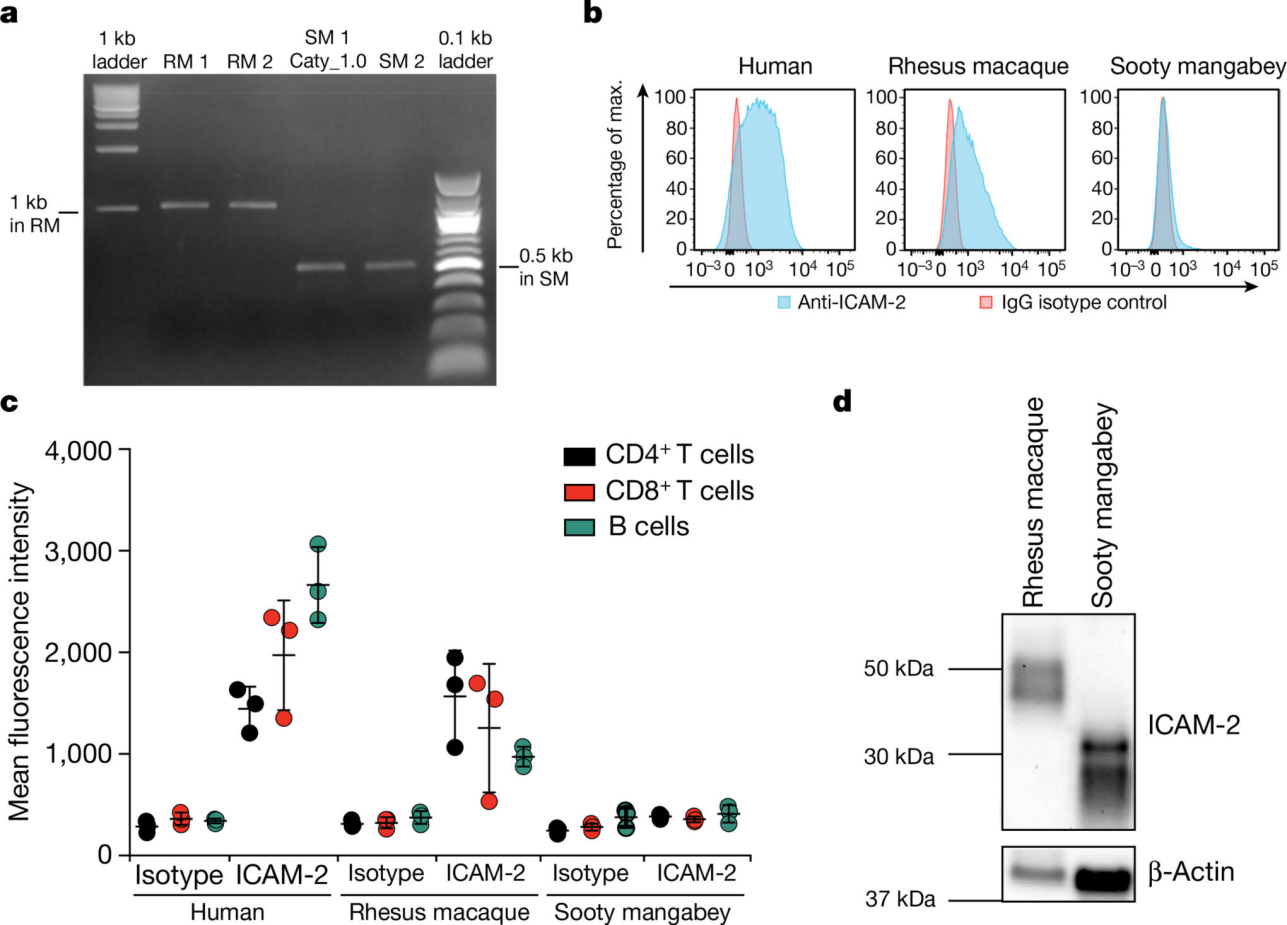
Genomic deletion in Ca*ICAM2* results in a truncated and dysfunctional protein **a**, PCR to confirm a putative 0.5-kb deletion in the Ca*ICAM2*. **b**, ICAM-2 surface expression of primary CD4^+^ cells by flow cytometry. *n* = 3; representative plots for **c**. **c**, ICAM-2 surface expression in B cells, CD4^+^ and CD8^+^ T cells from human, rhesus macaques and sooty mangabeys. *n* = 3 biologically independent samples for each species. **d**, ICAM-2-specific western blot using peripheral blood mononuclear cells from *M. mulatta* and *C. atys*. *n* = 3 *M. mulatta*; *n* = 2 *C. atys*; one representative biological sample per species is shown. For gel source data, see Supplementary Figs 1, 3.

The amino acid sequences of 9,257 *C. atys* proteins with RNA-seq support (Fig. 1) were aligned to *M. mulatta* orthologues as described above. We then used the codeml package from PAML (v.4.9a) on each of these alignments with the WAG amino acid rate matrix to calculate maximum likelihood genetic distances between the two sequences^40^. A histogram was generated from these distances with R (Extended Data Fig. 1a).

### *TLR4* gene tree

*TLR4* nucleotide sequences for 17 primate species were obtained from the NCBI GenBank resource (human: NM_138554.4; rhesus macaque: XM_015116960.1; sooty mangabey: manually curated XM_012091593.1; bonobo: NM_001279223.1; Nancy Ma’s night monkey: XM_012472756.2; drill: XM_011973281.1; colobus monkey: XM_011950060.1; crab-eating macaque: NM_001319615.1; squirrel monkey: XM_003925187.2; baboon: XM_003911309.4; pig-tailed macaque: NM_001305889.1; marmoset: XM_017975811.1; gorilla: XM_004048514.2; chimpanzee: NM_001144863.1; orangutan: AB445642.1; African green monkey: XM_007968248.1; gibbon: XM_003264057.3). These sequences were aligned with PASTA2 and we then constructed a maximum likelihood gene tree with RAxML3, performing 100 bootstrap replicates^41,42^ (Extended Data Fig. 7). Finding low bootstrap support amongst nodes ancestral to sooty mangabey, drill and baboon, we counted the number of sites that were discordant with respect to the gene tree topology. That is, the number of sites in which baboon and *C. atys* share the same state and *C. atys* and drill share a different state with an outgroup species (one of the two other Old World monkeys).

### Sample collection and processing

Peripheral blood samples from SIV-negative rhesus macaques and SIV-negative sooty mangabeys were collected by venipuncture according to standard procedures at the Yerkes National Primate Research Center of Emory University and in accordance with US National Institutes of Health guidelines. Human blood samples were obtained from healthy donors at the Yerkes National Primate Research Center in accordance with Institutional Review Board protocol IRB0004582 and all relevant ethical regulations. Informed consent was obtained from all blood donors. Peripheral blood mononuclear cells (PBMCs) were isolated from whole blood using Ficoll density-gradient centrifugation.

### *In vitro* TLR-ligand stimulation assay

The assay used in this study is a modified version of the procedure previously described^43^. Ultrapure LPS (*Escherichia coli* 0111:B4) and monophosphoryl lipid-A (*Salmonella minnesota*) were purchased from Invivogen. Whole blood collected in EDTA vacutainers was diluted 1:4 with RPMI 1640 medium and 195 µl aliquots were transferred to 96-well, round-bottom micro-titre plates. Agonists were diluted in RPMI 1640 and 5 µl were applied to the wells at the following final concentrations: LPS, 1,000–10 ng ml^−1^; lipid-A, 10–1 µg ml^−1^. Suspensions were then mixed by pipet and incubated at 37 ° C, 5% CO_2_ for 4 h). After incubation, plates were centrifuged at 700 r.p.m. for 10 min, and 120 µl of cell-free supernatant was removed and stored at −80 °C until the assay was carried out. Each TLR ligand at a given concentration was performed in triplicate for each animal.

### Cytokine bead array (CBA)

Samples were obtained from sooty mangabeys and rhesus macaques housed at the YNPRC. Sooty mangabeys were naturally infected at the YNPRC and rhesus macaques had been infected previously with SIV_smm_ as previously described^19^. Supernatant levels of TNF and IL-6 were measured using the human inflammation CBA kit (BD Biosciences Immunocytometry Systems) according to the manufacturer’s instructions, with the modification that the sample volumes for supernatant, antibody-coupled bead mix and PE-conjugated detection antibody solution were all reduced to 25 µl instead of 50 µl^44^. After incubation, samples were washed with 2% paraformaldehyde in PBS, resuspended in 150 µl PBS, and analysed using a FACSCalibur flow cytometer (BD Biosciences Immunocytometry Systems). The average of triplicate cytokine measurements was used as the representative value for individual animals, and variations in cytokine levels between species groups were tested for statistical significance using unpaired *t*-tests in Prism 6.0. To quantify the level of *TLR4* mRNA, and to perform linear regression of TLR-signalling molecules with TNF and IL6 cytokine levels, in the LPS-stimulated blood samples in the longitudinal SIV_smm_-infected samples, we used microarray expression data from matched whole-blood samples; these data are available from the NCBI Geo database (accession GSE16147).

### Plasma viral load measurement

Quantification of SIV_smm_ plasma viral RNA levels were quantified using qPCR as described previously^45,46^.

### RNA-seq analysis of LPS-stimulated monocytes

RNA-seq analysis was conducted at the Yerkes Nonhuman Primate Genomics Core Laboratory (http://www.yerkes.emory.edu/nhp_genomics_core/). CD14^+^ monocytes were isolated from Ficoll-isolated PBMCs using CD14 MicroBeads according to the manufacturer’s instructions (Miltenyi Biotec). Subsequently, 0.4 × 10^6^ cells were stimulated for 6 h with 10 ng ml^−1^ LPS and then immediately lysed in 350 µl RLT buffer (Qiagen). RNA was purified using Micro RNEasy columns (Qiagen) and RNA quality was assessed using Agilent Bioanalyzer. Then, 10 ng of total RNA was used as input for mRNA amplification using 5′ template-switch PCR with the Clontech SMART-Seq v.4 Ultra Low Input RNA kit, according to the manufacturer’s instructions. Amplified mRNA was fragmented and appended with dual indexed barcodes using Illumina NexteraXT DNA Library Prep kits. Libraries were validated by capillary electrophoresis on an Agilent 4200 TapeStation, pooled and sequenced on an Illumina HiSeq 3000 using (100 bp paired-end reads) at an average read depth of 18 million. RNA-seq data were analysed by alignment and annotation to either the MacaM v.7.8.2 assembly of the Indian rhesus macaque genome (available at https://www.unmc.edu/rhesusgenechip/index.htm) or to the Caty_1.0 assembly^22^. Alignment was performed using STAR v.2.5.2b using the annotation as a splice junction and abundance estimation reference, and non-unique mappings were removed from downstream analysis^47^. Transcripts were annotated using both the MacaM and Caty 1.0 assemblies and annotation as described in the text. Transcript abundance was estimated internally in STAR using the algorithm of HT-Seq and differential expression analyses were performed using the DESeq2 packages^48,49^. To quantitatively compare the degree to which LPS treatment induced inflammatory gene expression between species, we used GSEA^50^. GSEA was performed using the desktop module available from the Broad Institute (https://www.broadinstitute. org/gsea/)^51^. Gene ranks for contrasts of LPS-treated versus untreated samples were calculated from the normalized expression tables using the signal-to-noise metric for each species separately. Ranked datasets contrasting LPS-treated versus untreated samples were tested for enrichment of the gene sets ‘HALLMARK_TNFA_SIGNALING_VIA_NFKB’ (M5890) and ‘HALLMARK_IL6_JAK_STAT3_SIGNALING’ (M5897) from the Molecular Signatures Database (http://www.broadinstitute.org/gsea/msigdb/index.jsp) using gene set permutation to test for statistical significance. Heat maps and other visualizations were generated using Partek Genomics software, v.6.6.

### *ICAM2* exon splice junction analysis

RNA-seq alignments from all 24 LPS-stimulated monocyte samples, and alignments derived from deep RNA-seq (over 50 million reads) from two samples derived from flow-sorted, purified, blood *C. atys* conventional dendritic cells (cDCs, defined as CD3^−^CD14^−^CD20^−^CD123^−^HLA-DR^+^CD11c^+^) that were prepared alongside the monocytes were examined for observed splicing. To provide additional depth, we also included RNA-seq data from two flow-purified *M. mulatta* ‘non-classical’ monocyte samples (defined as CD14^−^CD16^+^HLA-DR^+^NKG2^−^CD3^−^CD20^−^) and one *C. atys* sample from CD4^+^ T transitional memory cells (CD4^+^ TTM, defined as CD3^+^ CD4^+^CD8^−^CD45RA^−^CD95^+^CD28^+^CCR7^high^CD62L^−^CD14^−^CD16^−^CD20^−^). Reads from the alignment (BAM) files that mapped from 5 kb upstream to 5 kb downstream of the *ICAM2* loci were scanned by a custom Perl script that recorded evidence of splicing from the CIGAR field, and accumulated counts of reads supporting either splicing or read-through at each site. Splice site counts for all the samples were added together and compared to find the proportion of reads supporting each splice variant or intronic retention.

### NF-κB luciferase reporter assay

Protein expressing constructs encoding human *TLR4*, Mm*TLR4*, Ca*TLR4*, Mm*TLR4* with the C terminus of Ca*TLR4*, and Ca*TLR4* with the C terminus of Mm*TLR4* were generated by the Emory Custom Cloning Core Division using standard cloning techniques. HEK293T were obtained from ATCC and regularly checked for mycoplasma contamination.

To determine the responsiveness of *Mm*TLR-4 and *Ca*TLR-4 to LPS, HEK293T cells were seeded in poly-l-lysine-coated 96-well plates and transfected in triplicate using a standard calcium phosphate transfection protocol. Cells were co-transfected with expression plasmids of human MD-2 (pEFBOS, 5 ng), human CD14 (pcDNA3, 5 ng) and different TLR-4 orthologues or chimaeras (pEF1a, 2.5 ng). The MD-2- and CD14-expression plasmids were provided by A. Medvedev; the NF-κB reporter construct was made available by B. Baumann^52,53^. A firefly-luciferase reporter under the control of three NF-κB-binding sites (100 ng) and a *Gaussia* luciferase reporter (5 ng) under the control of the pTAL promoter were co-transfected to monitor NF-κB activity. The pTAL promoter construct contains a minimal TATA-like promoter (pTAL) region from the herpes simplex virus thymidine kinase (HSV-TK) promoter (Clontech) that is nonresponsive to NF-κB and served as an internal control. To activate NF-κB, cells were stimulated with 5 µg ml^−1^ LPS (*E. coli* 026:B6, eBioscience) for 5 h. After 40 h of transfection, a dual luciferase assay was performed and the firefly luciferase signals were normalized to the corresponding *Gaussia* luciferase control values.

### qPCR

TLR stimulations of whole blood for qPCR were performed using the same method as for cytokine protein assay but scaled proportionally to use 1 ml of blood as input. Following stimulation, leukocytes were recovered by centrifugation at 700 r.p.m. for 5 min and removal of erythrocytes by incubation in ACK lysis buffer. Cells were lysed in 350 µl of RLT buffer, and RNA purified using the RNeasy Mini kit (Qiagen) according to the manufacturer’s instructions. qPCR was performed on RNA as previously described^54^. Primers to cytokines for qPCR were designed using Primer Express software (Applied Biosystems) to regions of 100% nucleotide identity between *M. mulatta* and *C. atys*: 12S rRNA (endogenous standard) forward 5′-CCCCCTAGAGGAGCCTGTTC-3′, 12S rRNA reverse 5′-GGCGGTATATAGGCTGAGCAA-3′; *TNF* forward 5′-GCCCTGGTATGAGCCCATCTA-3′, *TNF* reverse 5′-CGAGATAGTCGGGCA GATTGA-3′; *IL6* forward 5′-GAGAAAGGAGACATGTAACAGGAGTAAC-3′, *IL6* reverse 5′-TGGAAGGTTCAGGTTGTTTTCTG-3′. Fold change was calculated by dividing the normalized post-treatment sample quantity with the normalized untreated control quantity from the same animal, and calculating the average of fold changes for each species.

### Flow cytometry of PBMCs

Multicolour flow cytometry staining was performed using the following antibodies and reagents: CD3–APC/Cy7 (SP34-2), CD14–PE/Cy7 (M5E2) and CD20–PE/Cy5 (2H7) from BD; CD4–BV650 (OKT4), CD8–BV711 (RPA-T8), ICAM-2–FITC (CBR-IC2/2), Mouse IgG2a(κ)–FITC (MOPC-173) isotype control from Biolegend; Live/Dead Fixable Aqua from Thermo Fisher Scientific. Cells were stained for flow cytometry and data were acquired on an LSR II cytometer (BD) and analysed by FlowJo 10 software (TreeStar). Further analyses were performed using PRISM (GraphPad) and Excel (Microsoft Office 2011) software.

### ICAM-2 western blot

PBMCs were lysed in RIPA buffer and equal amounts of cell lysate were boiled after addition of sample buffer including β-mercaptoethanol, resolved with a 4–15% SDS–PAGE (Bio-Rad), and proteins were transferred to an Immobilon-P PVDF membrane (Millipore). Afterwards membranes were blocked for 1 h in blocking buffer (Bio-Rad) and incubated overnight with polyclonal rabbit ICAM-2-specific antibody (Bethyl). After washing (PBS with 0.05% Tween-20), anti-rabbit HRP-conjugated secondary antibody was incubated for an additional 1 h, washed, and HRP activity was determined using the Super Signal West Pico Kit (Bio-Rad and visualized using the ChemiDoc XRS+ (Bio-Rad). Then the membrane was stripped with buffer (2% SDS, 0.5 M Tris, pH 2.2), blocked again and β-actin was detected using a rabbit anti-β-actin antibody as primary antibody and anti-rabbit-HRP antibody as secondary antibody.

### Statistical analysis

Statistical significance was determined using an unpaired Student’s *t*-test with Welch’s correction. *P* < 0.05 was considered significant. * *P* < 0.05; ** *P* < 0.01; NS, not significant. Data are mean ± s.d. or s.e.m. as indicated. Significance for comparisons of mRNA levels of individual genes in RNA-seq data was tested using the Wald test as part of the DESeq2 workflow. Bars represent group means, and dots represent read counts for individual samples normalized to library size. *P* values denoted are adjusted using Benjamini–Hochberg correction.

### Code availability

We used a custom script to quantify ICAM-2 splice junctions. This script is available at Github: https://github.com/BosingerLab/splicing-analysis.

### Data availability

Raw sequences of the *C. atys* reference genome have been deposited in NCBI under Bioproject accession number PRJNA157077. The genome assembly is available at NCBI as Caty1.0 (RefSeq accession GCF_000955945.1). The multi-tissue *C. atys* RNA-seq reads are available from the Nonhuman Primate Reference Transcriptome Resource (NCBI SRA accession numbers SRX270666 and SRX270667). Data from Sanger sequencing of *TLR4* and *ICAM2* are available at NCBI (accession numbers MF468275–MF468286). Microarray data used for TLR-4 measurement and linear regression with TNF and IL-6 are available from the NCBI GEO database (accession GSE16147). The RNA-seq data for LPS-stimulated monocytes was submitted to the GEO database (accession numbers GSM2711028–GSM2711051 and GSE101617).

## Extended Data

**Extended Data Figure 2:**
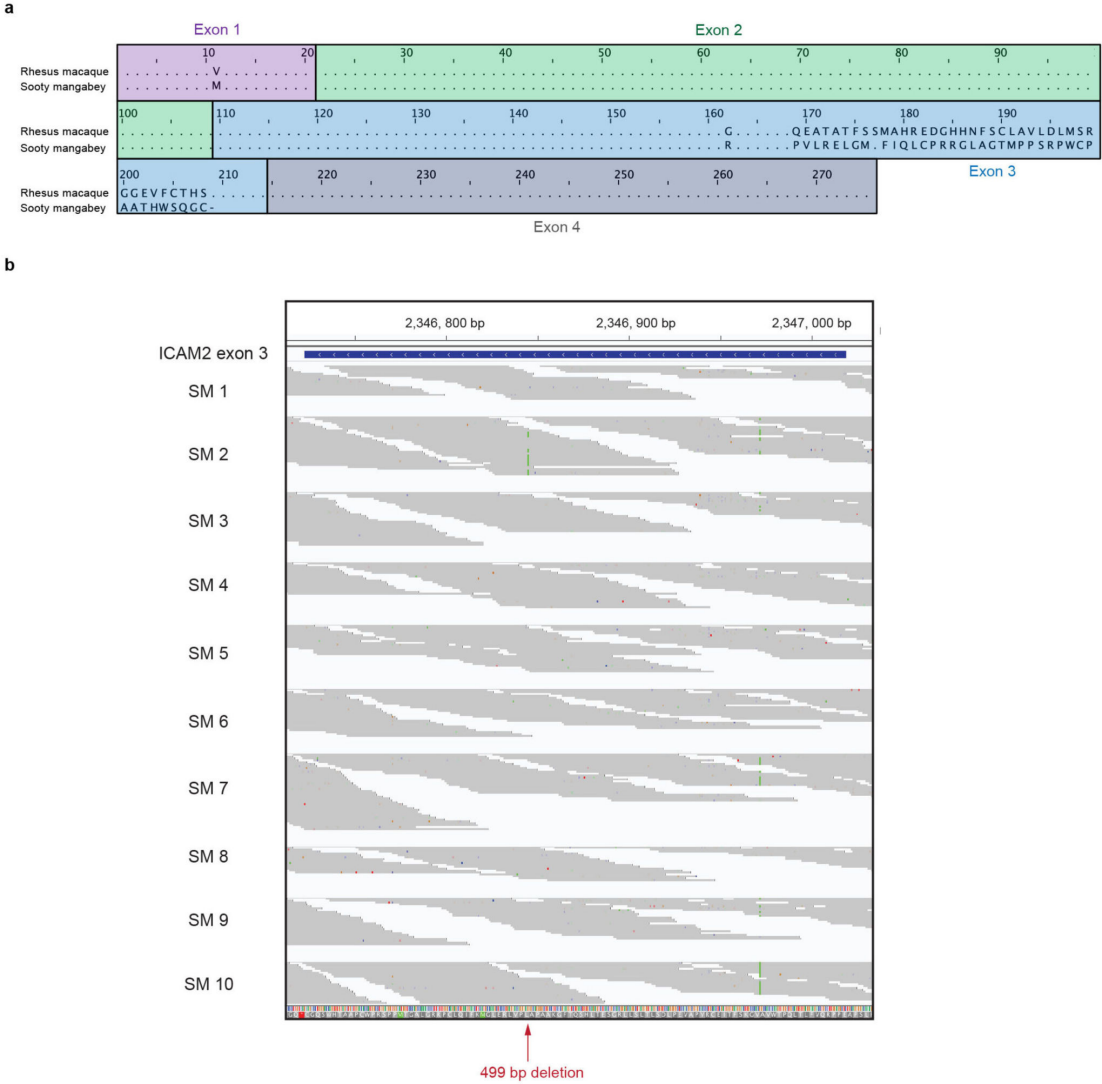
Sequence alignment of ICAM-2 protein and exon sequence analysis of *ICAM2* **a**, Pairwise alignment of predicted ICAM-2 protein models for sooty mangabey and rhesus macaque. Exon structure is highlighted based on human ICAM-2. Alignment was performed using Jalview v.2.9.0. **b**, The sequence of exon 3 of Ca*ICAM2* was confirmed in 10 additional individuals. Sequencing reads were aligned to the *C. atys* reference genome and visualized using Integrative Genomics Viewer (IGV). The red arrow indicates the position of the 499-bp genomic deletion in *C. atys*.

**Extended Data Figure 3:**
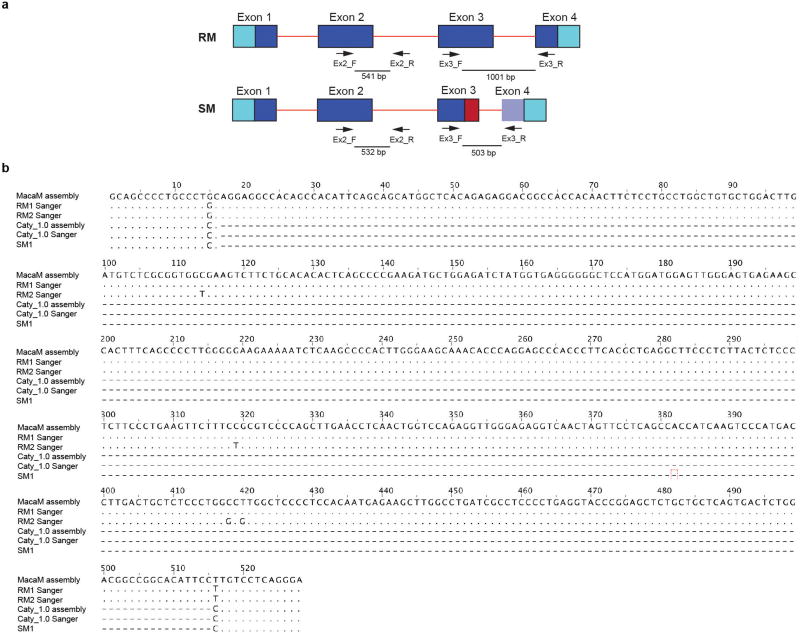
Predicted model of the *ICAM2* gene structure and *ICAM2* genome sequence alignments **a**, Predicted model of *ICAM2* gene structure of *M. mulatta* and *C. atys* and the location of PCR primers for Sanger sequencing. Light blue, untranslated region; dark blue, CDS; red lines, intronic sequence; dotted line, exonic and intronic sequences present in human *ICAM2* and Mm*ICAM2* but not in Ca*ICAM2*; red box, the sequence that would be intronic in Mm*ICAM2*, but which is included in the exonic sequence of Ca*ICAM2*; light-purple box for Ca*ICAM2* exon 4 represents the fact that the exon 4 sequence in Mm*ICAM2* is present in Ca*ICAM2* but is not included in the Ca*ICAM2* CDS due to a stop codon in the Ca*ICAM2* exon 3. Primer positions are indicated by arrows. Predicted PCR products are indicated by thick lines. Primers Ex3_F and Ex3_R were designed to amplify a region spanning a putative genomic deletion which includes the 3′ region of Ca*ICAM2* exon 3 and intron 3. **b**, Alignment of *ICAM2* genomic sequences. Sanger sequencing of 2 rhesus macaques and 2 sooty mangabeys (including the Caty_1.0 reference animal) was performed to confirm the *ICAM2* genomic deletion specific to *C. atys*. Starting at Mm*ICAM2* nucleotide position 3166, sequences were aligned using Jalview v.2.9.0. Dashed lines denote the deletion in *C. atys*. RM, rhesus macaque; SM, sooty mangabey.

**Extended Data Figure 4:**
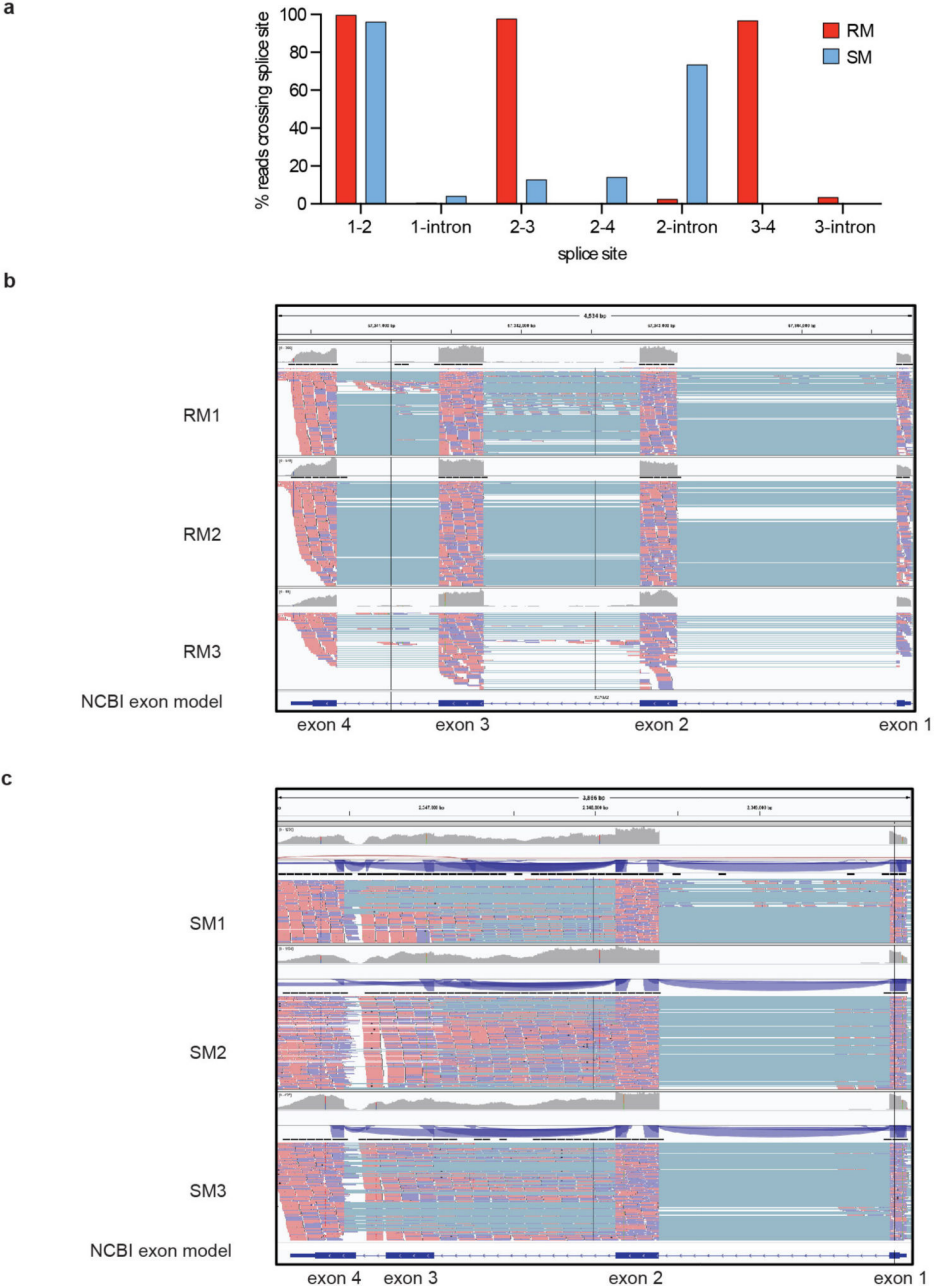
*ICAM2* splice junction analysis in *C. atys* and *M. mulatta* by RNA-seq read alignment **a**, Quantification of observed splicing. Splice site counts for RNA-seq read alignments were added together and sites with more than 100 total reads were compared to find the proportion of reads supporting each splice variant or intronic retention. **b**, Mm*ICAM2* splicing analysed by RNA-seq read alignment to the reference genome and visualized in IGV. **c**, Ca*ICAM2* splicing analysed by RNA-seq read alignment to the reference genome and visualized in IGV.

**Extended Data Figure 5:**
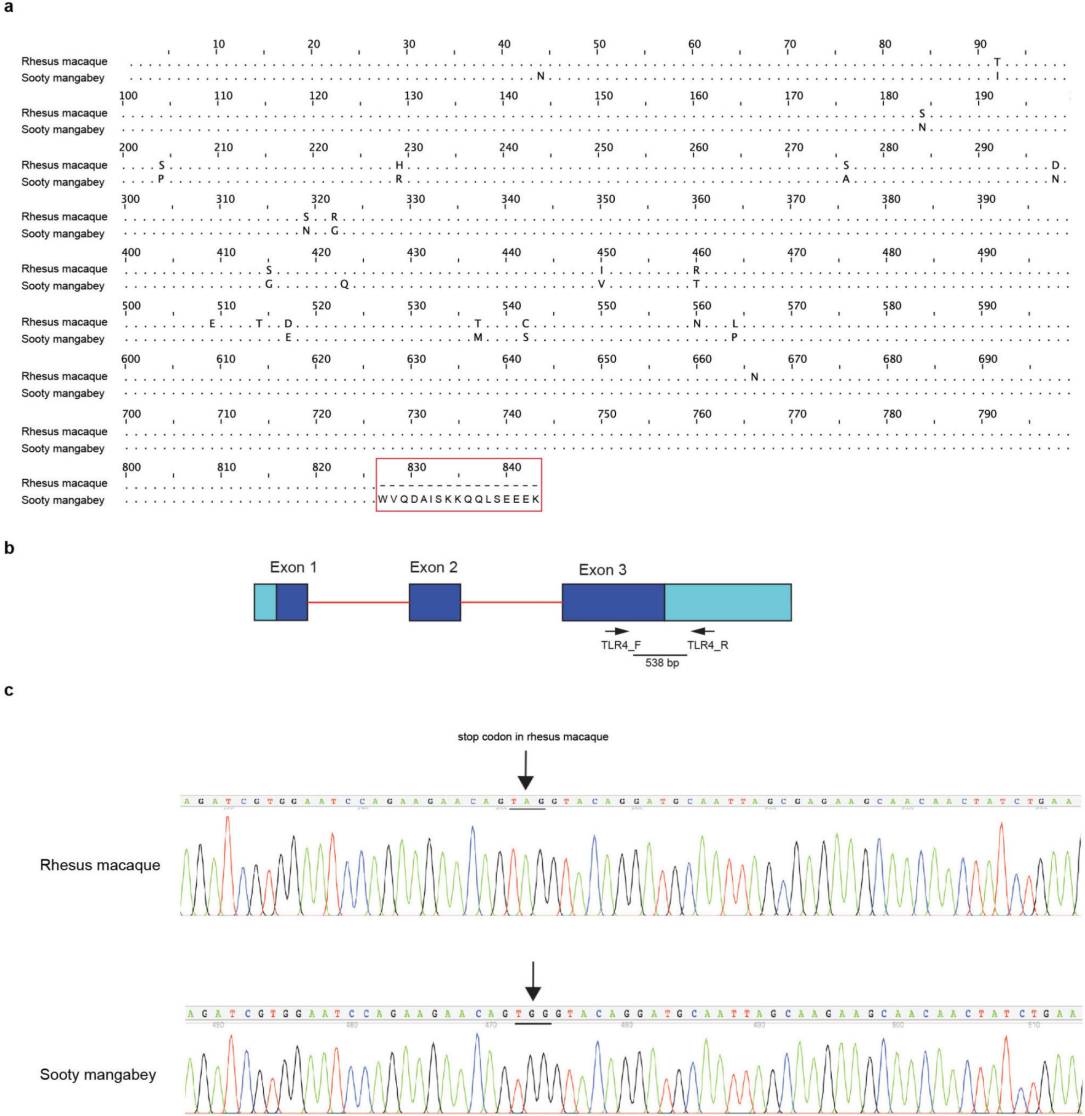
Sequence alignment of TLR-4 and the structure of the *TLR4* gene **a**, Pairwise alignment of TLR-4 protein sequences for *C. atys* and *M. mulatta*. The sequence difference at the C terminus is highlighted in red. Sequences were aligned using Jalview v.2.9.0. **b**, *TLR4* gene structure and location of PCR primers. Light blue, untranslated region; dark blue, CDS; red lines, intronic sequence. Primer positions are indicated by arrows. Predicted PCR product is indicated by thick line. Primers *TLR4*_F and *TLR4*_R were designed to amplify a region including a putative stop-loss mutation present in Ca*TLR4* but not in Mm*TLR4*. **c**, Chromatograms showing stop-loss (indicated by arrows) in the *TLR4* gene in *C. atys* with respect to *M. mulatta*. The relevant codon is underlined.

**Extended Data Figure 6:**
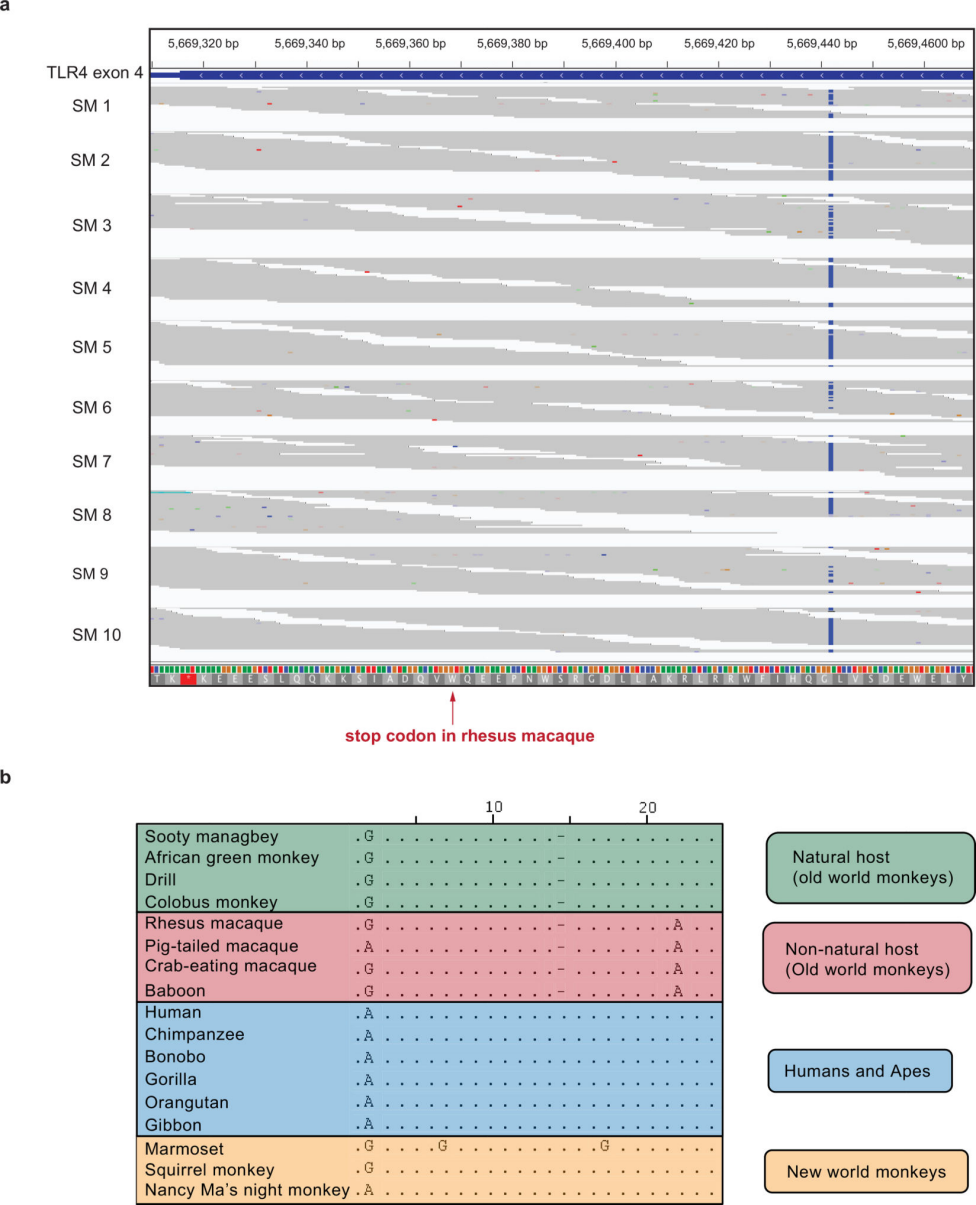
TLR-4 C terminus sequence aligments **a**, The sequence of the *Ca*TLR-4 C terminus was confirmed in 10 additional individuals. Sequencing reads were aligned to the Caty_1.0 reference genome and visualized in IGV. The red arrow indicates the position of the G-to-A stop codon mutation that can be found in Mm*TLR4* but not Ca*TLR4*. **b**, Alignment of genomic sequences encoding the TLR4 C terminus from different primate species. Starting at human *TLR4* nucleotide position 2461, sequences were aligned using Jalview v.2.9.0.

**Extended Data Figure 7:**
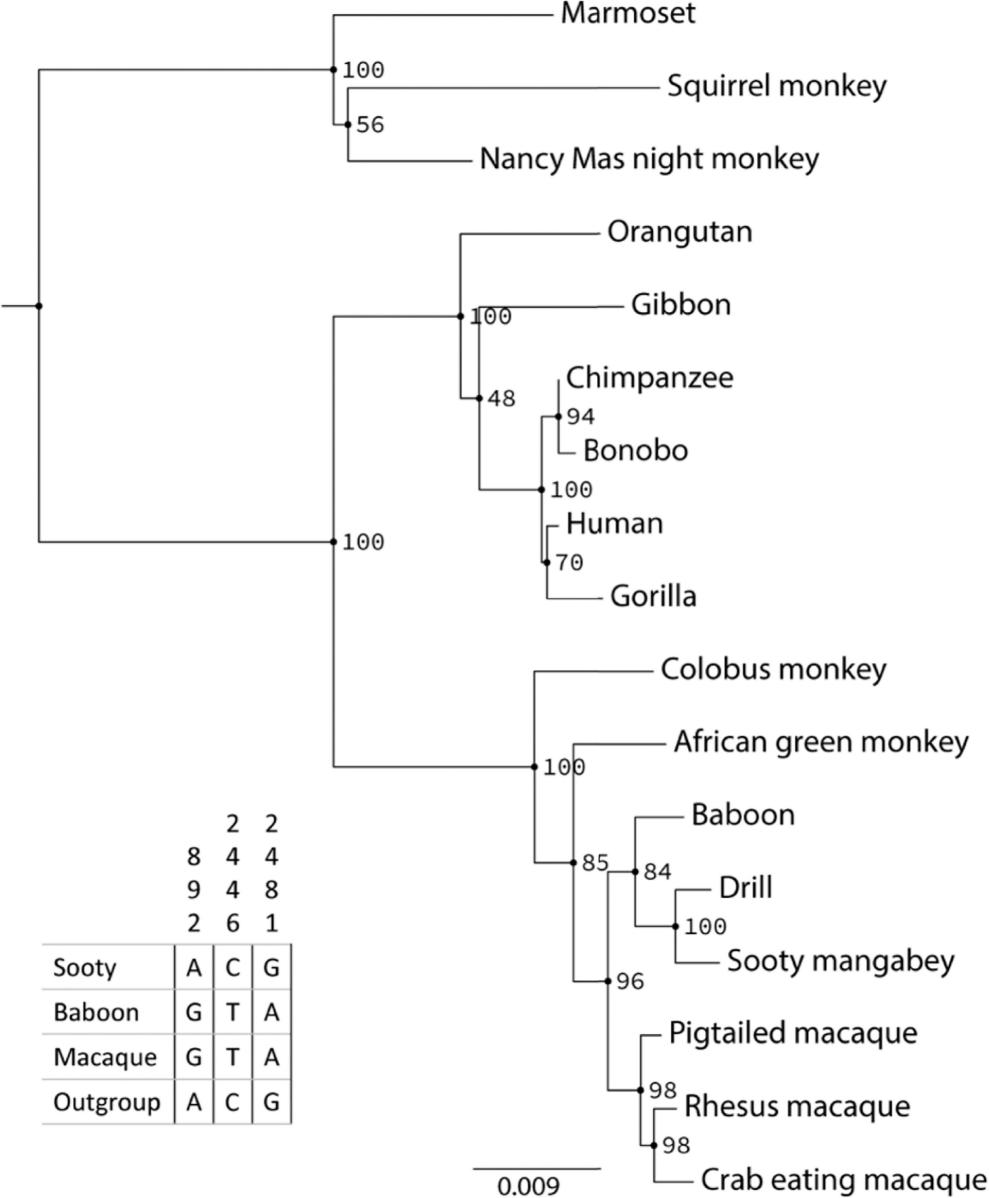
Maximum likelihood gene tree of *TLR4* This topology corresponds to the accepted species relationships for Old World monkeys. However, low bootstrap support among the nodes ancestral to *C. atys*, drill and baboon indicate that several sites within the gene do not support that ordering and may be indicative of incomplete lineage sorting. The table on the left shows these sites.

**Extended Data Figure 8:**
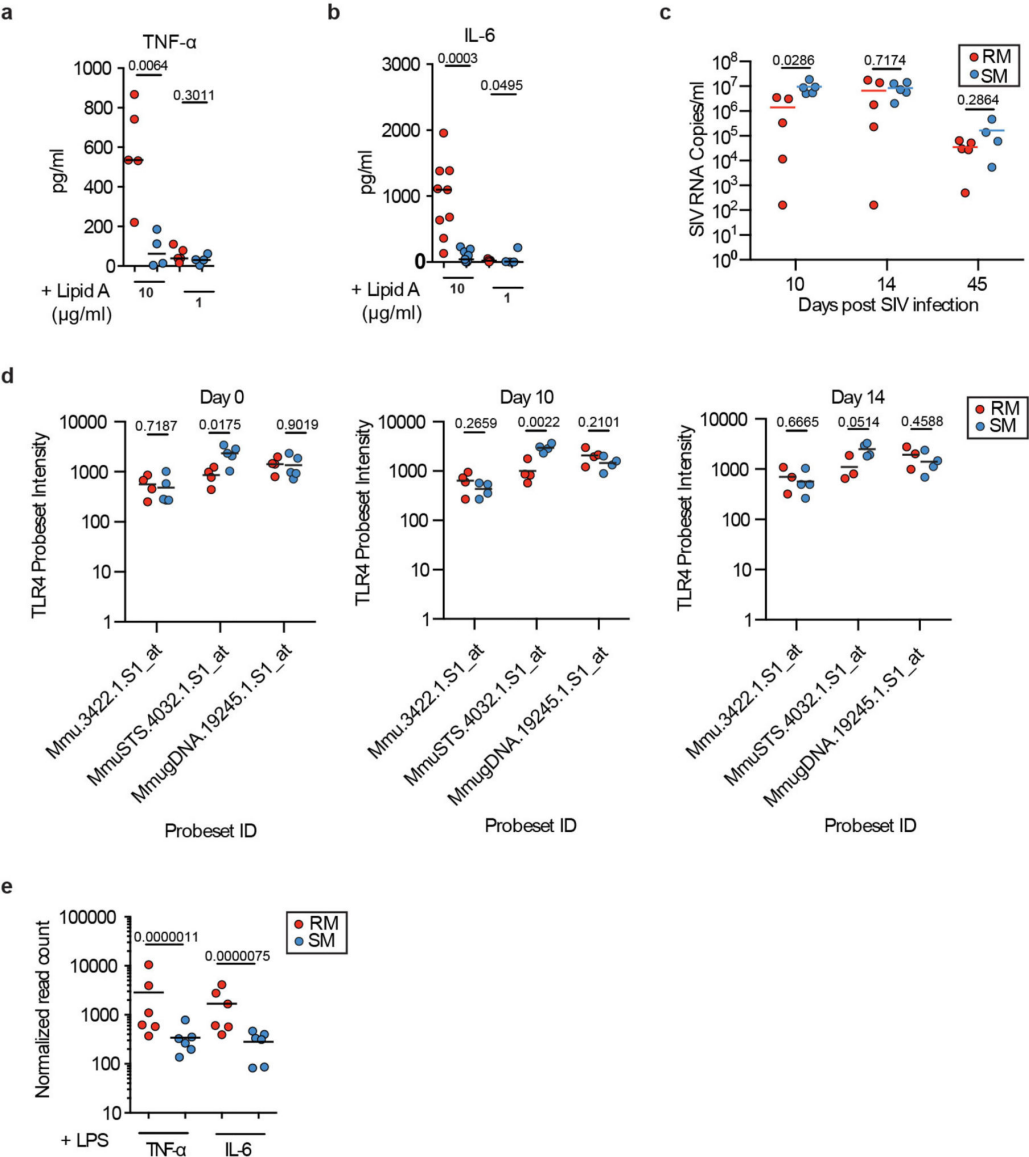
Analysis of cytokine expression and release after activation of TLR-4 **a**, TNF release from whole blood upon stimulation with lipid-A. Whole blood was stimulated with lipid-A at the indicated concentrations for 4 h and cytokine secretion was measured by cytometric bead array. *n* = 5 biologically independent samples for *M. mulatta*; *n* = 4 biologically independent samples for *C. atys*. **b**, IL-6 release from whole blood upon stimulation with lipid-A. Whole blood was stimulated with lipid-A at the indicated concentrations for 4 h and cytokine secretion was measured by cytometric bead array. *n* = 8 biologically independent samples for *M. mulatta*; *n* = 9 biologically independent samples for *C. atys*. **c**, SIV_smm_ plasma viral load for *M. mulatta* and *C. atys*. SIV_smm_ RNA levels in plasma were quantified at the indicated time points after intravenous inoculation with a primary uncloned SIV_smm_ *C. atys* isolate. *n* = 5 biologically independent samples for each species. **d**, *TLR4* mRNA levels in LPS-stimulated blood samples. To test the level of *TLR4* expression in the LPS-stimulated blood samples shown in Fig. 3e, we isolated RNA from whole blood from time-point matched replicate samples using PAXgene Blood RNA tubes, and analysed expression using Affymetrix GeneChip Rhesus Macaque Genome Arrays, which contains three independent probesets specific for Mm*TLR4* (denoted on the *x* axis). Probeset intensities are displayed along the *y* axis as RMA normalized values. *n* = 3 biologically independent samples for *M. mulatta*; *n* = 4 biologically independent samples for *C. atys*. **a**–**d**, Dots represent individual animals, and the bar represents the mean. Unpaired two-sided Student’s *t*-test, *P* values are indicated. **e**, *TNF* and *IL6* mRNA levels in LPS-stimulated monocytes from *M. mulatta* and *C. atys*. RNA-seq was used to assay global changes in gene expression after LPS stimulation of primary CD14^+^ monocytes. Significance for comparisons of mRNA levels of individual genes was tested using the Wald test as part of the DESeq2 workflow. Bars represent group means, and dots represent read counts for individual samples normalized to library size. Indicated *P* values are adjusted using the Benjamini–Hochberg correction. *n* = 6 biologically independent samples for each species.

**Extended Data Figure 9:**
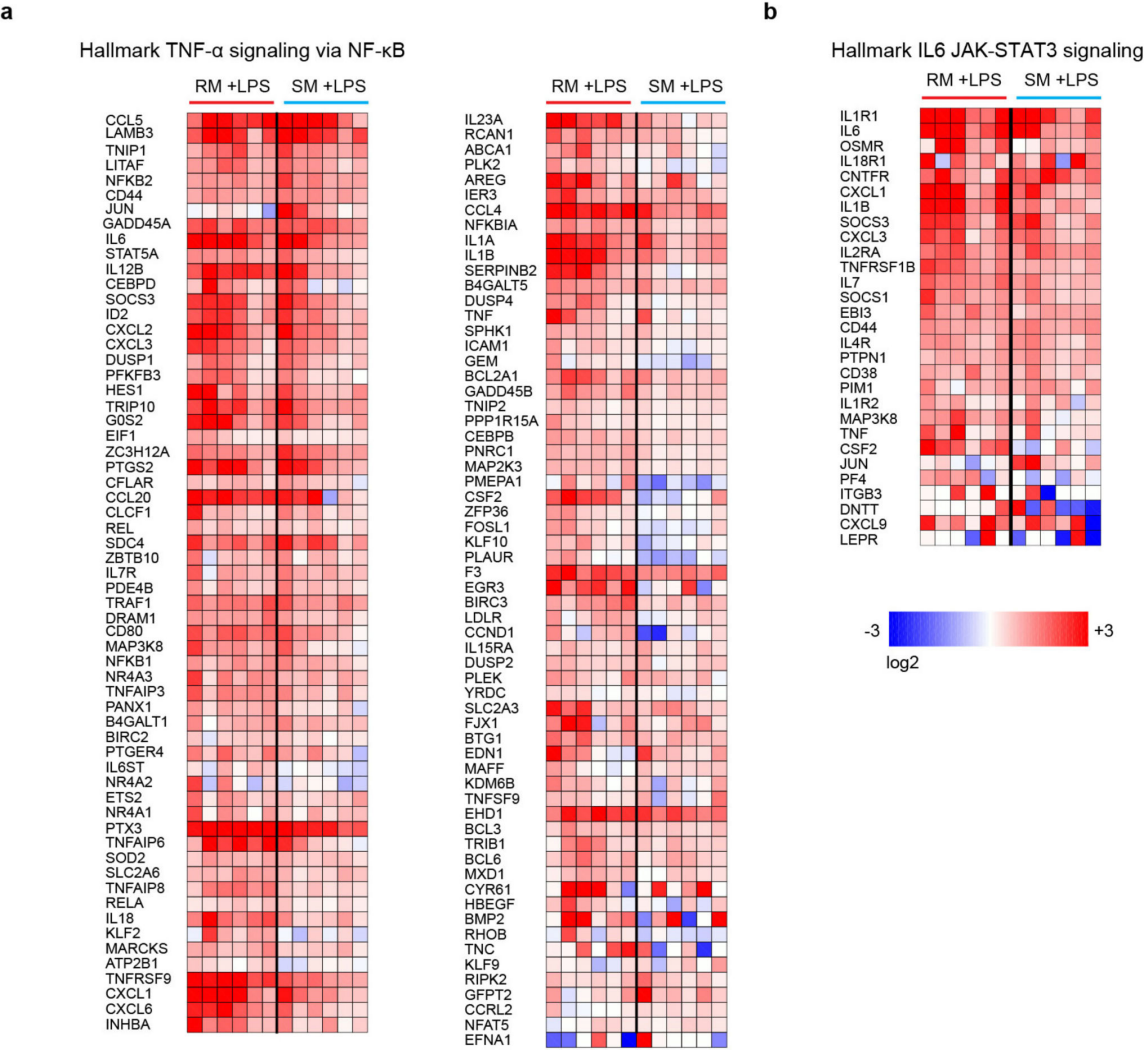
LPS-mediated induction of TNF and IL-6 inflammatory signalling is globally attenuated in *C. atys* **a**, **b**, Data shown are the leading-edge genes depicted in Fig. 3f, g (GSEA plots), for TNF-signalling genes (**a**) and IL-6-signalling genes (**b**). Values are the log_2_-transformed difference between LPS-treated and untreated samples for each individual animal. Genes selected are the combination of leading-edge/core-enriched genes for *M. mulatta* and *C. atys* GSEA analyses for each pathway. The gene sets selected for enrichment testing were obtained from the MSIGDB database hallmark collection are denoted at the top of each panel. Genes were organized using hierarchical clustering with Spearman dissimilarity and average linkage to estimate distance between genes and clusters, respectively. The colour scale at the bottom denotes the maximum and minimum on a log_2_ scale. For animal study source data, see Supplementary Table 2.

**Extended Data Table 1:** Amino acid divergence in proteins from *C. atys* identified by the immunogenomic comparison pipeline

Gene	Function	RM length (aa)	SM length (aa)	Identity (%)
CD24	B cell and granulocyte activation/differentiation	78	77	88.5
AP0BEC3C	retroviral restriction factor	190	190	91.6
DEFB129	antimicrobial	183	183	94.5
CLEC2D	inhibits NK-cell-mediated lysis	198	199	94.5
GZMA	cell lysis mediated by CD8+ T cells and NK cells	262	262	94.7
PGLYRP1	Peptidoglycan recognition on gram-positive bacteria	196	196	94.9
CCL24	chemoattractant for resting T cells	119	119	95.0
C5AR1	complement receptor	350	350	95.1
BST2	retroviral restriction factor	182	182	95.6
PF4	coagulation, chemoattractant for neutrophils and monocytes	196	196	96.0
S100A7	antimicrobial, immunomodulatory	101	101	96.0
CLEC6A	mannose-dependent pathogen recognition, proinflammatory	209	209	96.2
MB21D1	antiviral, cytosolic DNA sensor	522	522	96.2
BPI	antimicrobial, LPS-sensing	487	487	96.3
PRG3	cytotoxic and cytostimulatory activities	225	225	96.4
GSDMD	antimicrobial, pyroptosis	484	484	96.5
CLEC4A	Pattern recognition receptor	204	204	96.6
CLEC4D	inflammation and immune responses	215	215	96.7
PPBP	chemoattractant and activator of neutrophils	128	128	96.8
CD4	T cell receptor activation, HIV/SIV receptor	458	458	96.9
CTSG	lysosomal antigen processing	255	255	96.9
CD33	adhesion molecule on myeloid cells	359	359	96.9
LY96/MD2	associates with TLR4 for LPS binding	160	160	96.9
CCL11	chemoattractant for eosinophils	97	97	96.9

aa, amino acids.

**Extended data Table 2:** Analysis of immune gene families across species

Panel A								

Change Type	gene family	function	SM	AGM	RM	Human	Chimp	Baboon
Expansion (+5)	ADAM metalloproteinases	cytokine regulation	30	20	27	22	18	24
Expansion (+6)	scavenger receptors	LDL binding	17	9	11	9	10	10
Expansion (+6)	butyrophilin	lymphocyte deactivation	16	10	9	9	7	10
Expansion (+3)	TNFRSF10/TRAIL	apoptosis induction	6	4	3	4	5	3
Expansion (+2)	CD300	lipid-binding, immunomodulation	5	3	2	3	3	3

Contraction (−3)	C-C-motif chemokines	chemoattractant for immune cells	6	9	10	20	9	10

Panel B			

	*λ* (No Error Model)	*ε* (Estimated error)	*λ* (Error Model = *ε*)
10 species in this study	0.00268	0.04268	0.00204
11 species Gibbon	0.00258	0.04101	0.00141
Genome Project^50^			
10 mammal dataset^49^	0.00238	0.07324	0.00186

Panel C								

	Expansions			Contractions			No Change	Avg. Expansion

	Families	Genes gained	genes/ expansion	Families	Genes lost	genes/ contraction		
Sooty	535 (96)	1153	2.16	340 (48)	494	1.45	10106	0.024528
Human	1042 (276)	3471	3.33	192 (10)	210	1.09	9747	0.200967
Marmoset	1027 (122)	2213	2.15	668 (23)	841	1.26	9286	0.107504
Chimp	161 (23)	384	2.39	874 (69)	1137	1.3	9946	−0.081244
Gibbon	354 (13)	552	1.56	1089 (92)	1466	1.35	9538	−0.085529
Baboon	290 (61)	660	2.28	624 (41)	737	1.18	10067	−0.028084
Ora ng	548 (65)	1032	1.88	749 (14)	820	1.09	9684	−0.003921
Macaque	1101 (203)	2904	2.64	783 (22)	835	1.07	9097	0.100666
Mouse	631 (38)	2719	4.31	855 (9)	1027	1.2	9495	0.013404
Vervet	294 (19)	658	2.24	674 (59)	921	1.37	10013	−0.039209

a , Expansion and contraction of immune gene families across six primate species.

b , Assembly and annotation error estimations and gene gain and loss rates in a single *λ* model in 13 mammals.

c , Summary of gene gain and loss events inferred after correcting for annotation and assembly errors across all 13 species. The number of rapidly evolving families is shown in parentheses for each type of change.

AGM, African green monkey.

**Extended Data Table 3:** Correlation analysis between TLR-signalling molecules and gene expression

**Gene** **Name**	**Gene Symbol** **- RM**	**Affymetrix Probeset ID**	**r – SM** **TNF**	**p value –** **SM TNF**	**Lower** **CI SM**	**Upper** **CI SM**	**r - RM** **TNF**	**p-value -** **RM TNF**	**Lower** **CI RM**	**Upper** **CI RM**

AP1	JUN	MmugDNA.22829.1.S1_at	−0.52	0.07	−0.83	0.04	0.14	0.7	−0.54	0.71
CD14	CD14	MmuSTS.1982.1.S1_at	−0.28	0.35	−0.72	0.32	0.3	0.41	−0.41	0.78
IKKA	CHUK	MmuSTS.1867.1.S1_at	0.25	0.4	−0.35	0.71	−0.31	0.38	−0.79	0.40
IKKB	IKBKB	MmugDNA.8188.1.S1_at	−0.51	0.07	−0.83	0.05	0.05	0.88	−0.60	0.66
IKKG	IKBKG	MmuSTS.4600.1.S1_at	−0.26	0.4	−0.71	0.34	0.14	0.7	−0.54	0.71
IRAK1	IRAK1	MmugDNA.38816.1.S1_at	−0.33	0.28	−0.74	0.27	0.3	0.39	−0.40	0.78
IRF7	IRF7	MmugDNA.29625.1.S1_at	−0.6	0.03	−0.86	−0.07	−0.16	0.66	−0.72	0.52
JNK	MAPK8	MmugDNA.7819.1.S1_at	0.48	0.1	−0.10	0.81	−0.27	0.45	−0.77	0.43
MYD88	MYD88	MmugDNA.10008.1.S1_at	−0.41	0.17	−0.78	0.18	−0.08	0.83	−0.67	0.58
NFKB1	NFKB1	MmuSTS.3011.1.S1_at	−0.29	0.34	−0.73	0.31	0.2	0.58	−0.49	0.74
NFKB2	NFKB2	MmugDNA.25060.1.S1_at	−0.5	0.08	−0.82	0.07	0.07	0.86	−0.59	0.67
P38	MAPK1	MmugDNA.1694.1.S1_at	0.43	0.15	−0.16	0.79	0.09	0.8	−0.57	0.68
RIP1	RIPK1	MmugDNA.40799.1.S1_at	−0.36	0.23	−0.76	0.24	0.21	0.57	−0.49	0.74
TAB1	TAB1	MmuSTS.1553.1.S1_at	−0.15	0.63	−0.65	0.44	0.42	0.23	−0.29	0.83
TAK1	MAP3K7	MmugDNA.34734.1.S1_s_at	0.87	0	0.62	0.96	−0.3	0.4	−0.78	0.41
TBK1	TBK1	MmuSTS.3947.1.S1_at	−0.09	0.78	−0.61	0.49	−0.5	0.14	−0.86	0.19
TIRAP	TIRAP	MmugDNA.502.1.S1_at	−0.48	0.1	−0.81	0.10	0.1	0.79	−0.57	0.69
TLR4	TLR4	MmuSTS.4032.1.S1_at	−0.19	0.52	−0.67	0.40	−0.69	0.03	−0.92	−0.10
TRAF6	TRAF6	MmuSTS.4612.1.S1_at	0.48	0.09	−0.09	0.82	0.04	0.91	−0.60	0.65
TRAM	TICAM2	MmuSTS.930.1.S1_at	−0.24	0.43	−0.70	0.36	−0.6	0.07	−0.89	0.05
TRIF	TICAM1	MmugDNA.27425.1.S1_at	−0.35	0.23	−0.76	0.24	0.58	0.08	−0.08	0.88

**Gene** **Name**	**Gene Symbol** **- RM**	**Affymetrix Probeset ID**	**r -SM** **IL6**	**p-value -** **SM IL6**	**Lower** **CI SM**	**Upper** **CI SM**	**r - RM** **IL6**	**p-value** **RM IL6**	**Lower** **CI SM**	**Upper** **CI SM**

AP1	JUN	MmugDNA.22829.1.S1_at	−0.45	0.12	−0.80	0.13	0.23	0.52	−0.47	0.75
CD14	CD14	MmuSTS.1982.1.S1_at	−0.05	0.87	−0.58	0.52	0.01	0.98	−0.62	0.64
IKKA	CHUK	MmuSTS.1867.1.S1_at	−0.02	0.95	−0.56	0.54	0.05	0.89	−0.60	0.66
IKKB	IKBKB	MmugDNA.8188.1.S1_at	0.07	0.82	−0.50	0.60	0 12	0.73	−0.55	0.70
IKKG	IKBKG	MmuSTS.4600.1.S1_at	−0.25	0.4	−0.71	0.35	−0.04	0.91	−0.65	0.60
IRAK1	IRAK1	MmugDNA.38816.1.S1_at	−0.24	0.43	−0.70	0.36	−0.22	0.54	−0.75	0.47
IRF7	IRF7	MmugDNA.29625.1.S1_at	0.05	0.88	−0.52	0.58	−0.04	0.91	−0.66	0.60
JNK	MAPK8	MmugDNA.7819.1.S1_at	−0.01	0.97	−0.56	0.54	0.32	0.37	−0.39	0.79
MYD88	MYD88	MmugDNA.10008.1.S1_at	−0.09	0.77	−0.51	0.48	0.34	033	−0.35	0.80
NFKB1	NFKB1	MmuSTS.3011.1.S1_at	−0.05	0.87	−0.59	0.51	0.37	0.3	−0.34	0.81
NFKB2	NFKB2	MmugDNA.25060.1.S1_at	−0.01	0.97	−0.56	0.54	−0.32	0.36	−0.79	0.38
P38	MAPK1	MmugDNA.1694.1.S1_at	−0.25	0.41	−0.70	0.35	−0.15	0.69	−0.71	0.53
RIP1	RIPK1	MmugDNA.40799.1.S1_at	−0.61	0.03	−0.87	−0.10	0.34	0.34	−0.37	0.80
TABt	TAB1	MmuSTS.1553.1. S1_at	−0.31	0.3	−0.74	0.29	−0.26	0.47	−0.76	0.44
TAK1	MAP3K7	MmugDNA.34734.1.S1_s_at	0.03	0.91	−0.53	0.57	−0.31	0.38	−0.79	0.39
TBK1	TBK1	MmuSTS.3947.1.S1_at	−0.11	0.72	−0.62	0.47	0.35	0.32	−0.36	0.80
TIRAP	TIRAP	MmugDNA.502.1.S1_at	−0.06	0.83	−0.59	0.50	0.35	0.33	−0.36	0.80
TLR4	TLR4	MmuSTS.4032.1.S1_at	−0.13	0.68	−0.63	0.46	−0.15	0.68	−0.71	0.53
TRAF6	TRAF6	MmuSTS.4612.1.S1_at	0.58	0.04	0.05	0.86	0.25	0.49	−0.45	0.76
TRAM	TICAM2	MmuSTS.930.1.S1_at	0.14	0.65	−0.45	0.64	−0.17	063	−0.72	0.51
TRIF	TICAM1	MmugDNA.27425.1.S1_at	−0.25	0.41	−0.70	0.35	0.05	0.89	−0.60	0.66

Pearson’s correlation coefficients (*r*) were calculated separately for cytokines from *C. atys* and *M. mulatta* (TNF or IL-6) protein measurements versus mRNA levels of TLR-4-signalling genes measured in matched blood samples using Affymetrix GeneChips. *P* values denote the significance of the Pearson’s correlation coefficient. CI, confidence interval.

## Supplementary Material

Supplementary Information

## Figures and Tables

**Figure 3:**
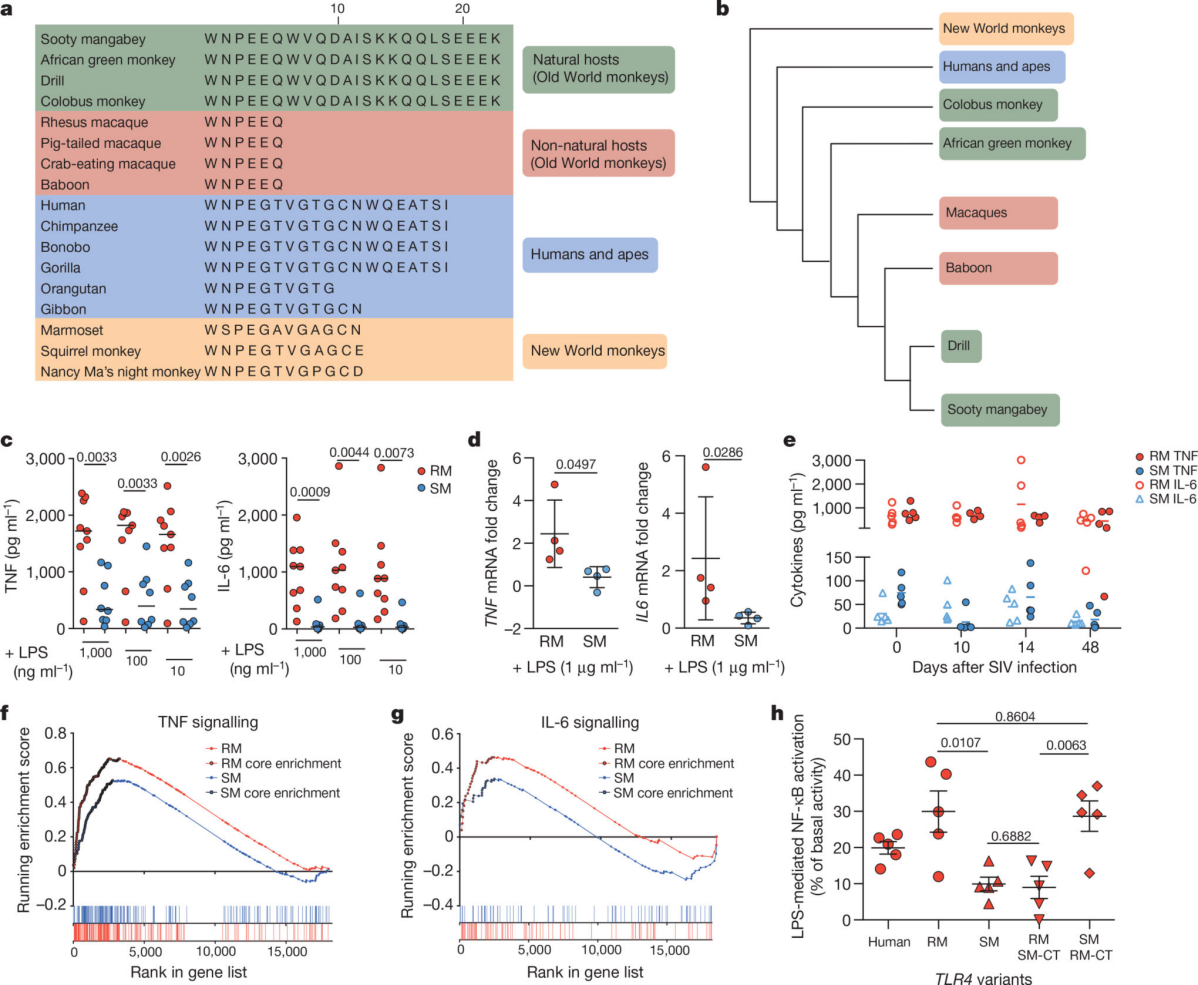
The TLR-4 C terminus is distinctive in natural SIV hosts **a**, Alignment of C-terminal TLR-4 protein sequences from different primate species (starting at human TLR-4 amino acid position 821). **b**, Primate phylogenetic tree with colour-coding according to the TLR-4 C terminus as indicated in **a**. Phylogeny appears as in ref. 14. **c**, Cytokine release from blood of rhesus macaques (*n* = 9 biologically independent samples) and sooty mangabeys (*n* = 8 biologically independent samples) after LPS stimulation as measured by cytometric bead array. **d**, mRNA expression in whole blood after LPS stimulation quantified by quantitative PCR (qPCR). *n* = 4 biologically independent samples for each species. **e**, TNF and IL-6 cytokine release from blood of rhesus macaques and sooty mangabeys over the course of SIV infection. *n* = 5 biologically independent samples for each species. Data are mean ± s.d. (**c**–**e**), unpaired two-sided Student’s *t*-test, *P* values are indicated (**c**, **d**). **f**, Gene set enrichment analysis of LPS-stimulated monocytes of rhesus macaques and sooty mangabeys using the TNF signalling via NF-κB hallmark gene set. **g**, GSEA of LPS-stimulated monocytes of rhesus macaques and sooty mangabey using the IL6 JAK–STAT3 hallmark gene set. **h**, NF-κB response to LPS of primate *TLR4* variants in transfected HEK293T cells. NF-κB firefly-luciferase signals were normalized to *Gaussia* luciferase signals, and the relative increase in NF-κB activity compared to unstimulated controls (100%) was calculated. Data are mean ± s.e.m. of *n* = 5 independent experiments performed in triplicate transfections are shown. Unpaired two-sided Student’s *t*-test, *P* values are indicated. For source data of the animal studies, see Supplementary Table 1. RM SM-CT, *Mm*TLR-4 with the C terminus of *Ca*TLR-4; SM RM-CT, *Ca*TLR-4 with the C terminus of *Mm*TLR-4.

**Table 1:** *C. atys* assembly statistics and proteins with major structural variations in the *C. atys* genome

Assembly		Annotation	
Average coverage per base	192	Protein-coding genes	20,829
Total sequence length	2,848,246,356 bp	Non-coding genes	4,464
Total assembly gap length	60,973,502 bp	Pseudogenes	5,263
Number of scaffolds	11,433	mRNA transcripts	65,920
Scaffold N50	12,849,131 bp	lncRNA transcripts	6,299
Scaffold L50	66	Exons in coding transcripts	250,660
Number of contigs	76,752	Exons in non-coding transcripts	42,280
Contig N50	112,942 bp		
Contig L50	6,930		
GC content	40.90%		

**Gene**	**Function**	**Variation type**	**Length variation (amino acids)**

*ICAM2*	Lymphocyte extravasation and recirculation	indel, fs	107
*TLR4*	LPS sensing	indel, fs	17
*BPIFA1*	Antimicrobial function in airways	indel	8
*NOS2*	Proinflammatory messenger	pm, early stop	8
*MBL2*	Pattern recognition receptor for microbial products	pm, early start	7
*TREM2*	Chronic proinflammatory signalling in myeloid cells	indel, fs	6
*PLSCR1*	Enhancement of the interferon response	indel	5
*LST1*	Inhibition of lymphocyte proliferation	indel, fs	5
*CRTAM*	T and natural killer cell activation	pm, indel	4

Structural variations were identified by the immunogenomic comparison pipeline. N50, 50% of the genome is in fragments of this length or longer; L50, smallest number of fragments needed to cover more than 50% of the genome; lncRNA, long non-coding RNA; indel, insertion/deletion; fs, frameshift; pm, point mutation.
