# Supplementary material for: Sooty mangabey genome sequence provides insight into AIDS resistance in a natural SIV host
Source: Nature. Author manuscript; Available in PMC 2018 Apr 3. (PMC5843367; doi:10.1038/nature25140)
Supplement: Supplementary Information [file NIHMS941013-supplement-Supplementary_Information.pdf]

Supplementary Figure 1.
(Gel source data for Figure 2a.)

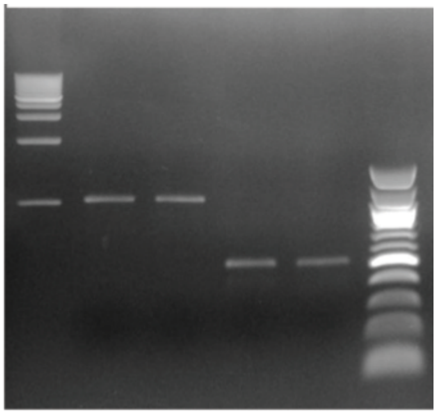

Supplementary Figure 2.
(Gating strategy for figure 2 b, c)

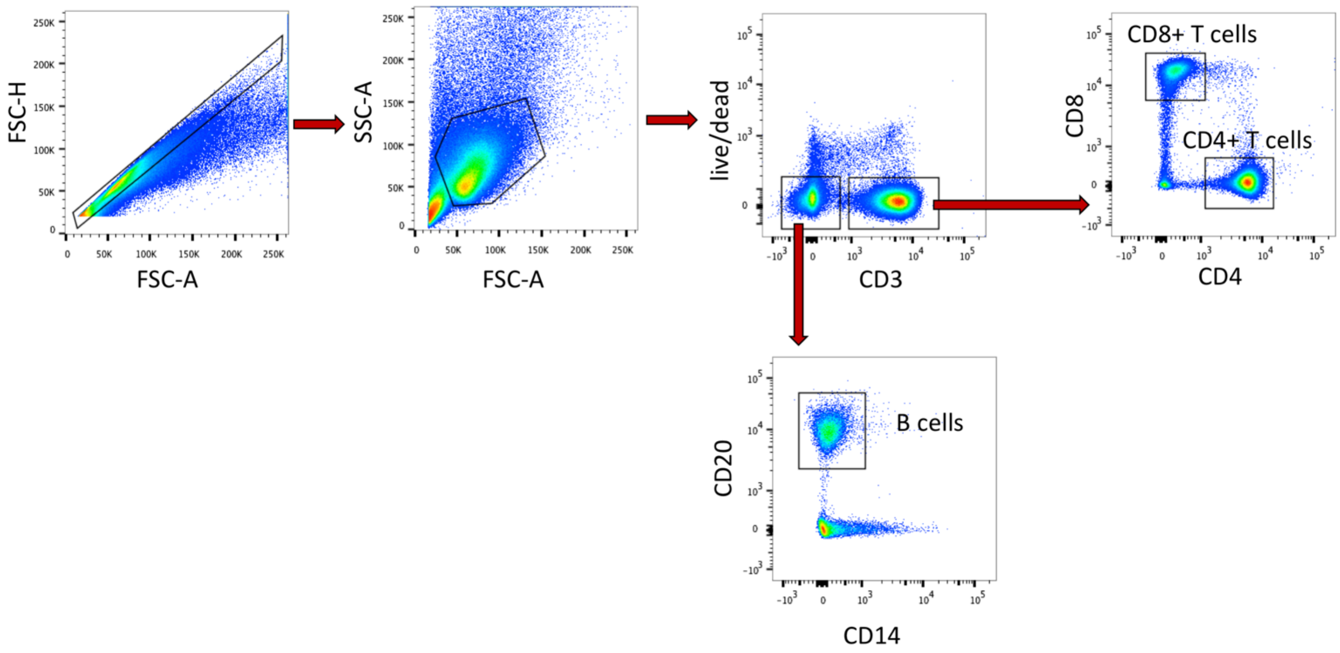

Supplementary Figure 3.

All three images (a, b, c) depict the same Western blot (Gel source data for Fig. 2d).

a) Anti-ICAM2 staining

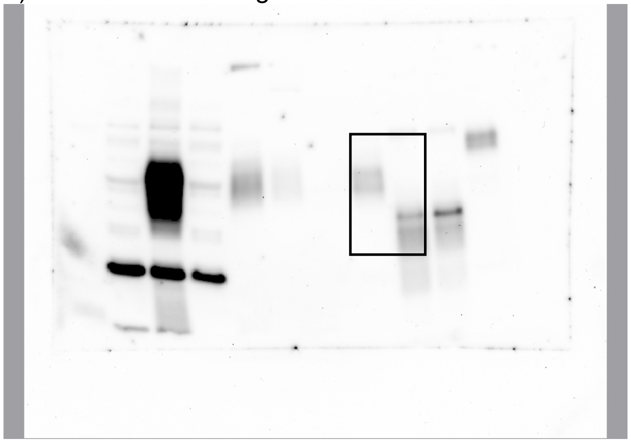

b) Anti-β-actin staining

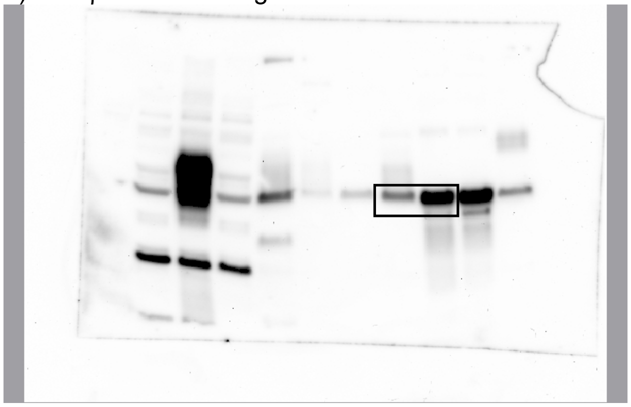

c) Exposure to visualize protein standard

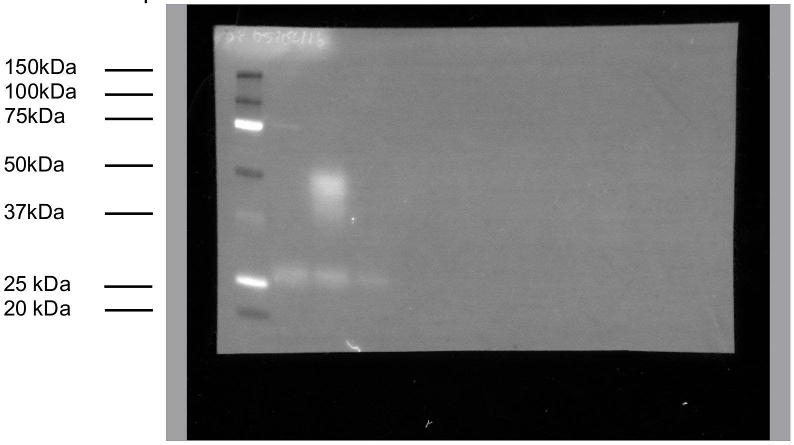

Supplementary table 1.
(source data for animal study data in figure 2 e)

weeks (p.i.)	RM TNF-α (pg/ml)					SM TNF-α (pg/ml)					RM IL6 (pg/ml)					SM IL6 (pg/ml)				
0	484	608	803	1297	762	118	50	86	53	67	784	1242	316	654	400	75	24	18	24	15
10	520		781	652	895	3	55	1	2	2	1112		400	572	603	102	50	19	26	21
14	605	679	606	663	387	90	24	38	138	38	1940	3011	380	265	176	53	63	17	83	12
48	170	67	337	846	856	4	7	47	1	33	587	752	122	390	476	30	11	10	8	11

RM=rhesus macaque, SM=sooty mangabey, pi.i.= post infection

Supplementary table 2.
(source data for animal study data in Extended data figure 8 c)

weeks p.i.	RM SIVsmm (SIV RNA copies/ml)					SM SIVsmm (SIV RNA copies/ml)				
10	3530000	160	11800	331000	3130000	18900000	5020000	8820000	5420000	9500000
14	14000000	160	233000	1820000	17800000	14400000	5810000	2020000	7360000	12600000
45	63700	508	29800	27800	51400	61400	140000		468000	5380

RM=rhesus macaque, SM=sooty mangabey, pi.i.= post infection
